# A paradigm shift in all-solid-state lithium batteries: halide cathode materials streamlined for multi-electron reactions

**DOI:** 10.1093/nsr/nwag438

**Published:** 2026-07-16

**Authors:** Yang Luo, Yuhao Duan, Xiaofei Yang, Xueliang Sun, Xianfeng Li

**Affiliations:** School of Energy and Environmental Engineering, Hebei University of Technology, Tianjin 300401, China; Dalian Institute of Chemical Physics, Chinese Academy of Sciences, Dalian 116023, China; School of Chemical Engineering, University of Chinese Academy of Sciences, Beijing 100049, China; Dalian Institute of Chemical Physics, Chinese Academy of Sciences, Dalian 116023, China; Key Laboratory of Long-Duration and Large-Scale Energy Storage, Dalian Institute of Chemical Physics, Chinese Academy of Sciences, Dalian 116023, China; Eastern Institute for Advanced Study, Eastern Institute of Technology, Ningbo 315200, China; Dalian Institute of Chemical Physics, Chinese Academy of Sciences, Dalian 116023, China; Key Laboratory of Long-Duration and Large-Scale Energy Storage, Dalian Institute of Chemical Physics, Chinese Academy of Sciences, Dalian 116023, China; Dalian National Laboratory for Clean Energy, iChEM (Collaborative Innovation Center of Chemistry for Energy Materials), Dalian Institute of Chemical Physics, Chinese Academy of Sciences, Dalian 116023, China

**Keywords:** halide cathodes, all-solid-state lithium batteries, ion/electron conduction, single-phase interface, multi-electron reaction mechanisms

## Abstract

Halide cathode active materials (CAMs) are emerging as a transformative platform for all-solid-state lithium batteries (ASSLBs), offering intrinsic high ionic/electronic conductivities, multi-electron transfer capability and the unique potential for single-phase electrode architectures that eliminate inactive components. Despite these advantages, critical challenges regarding their reaction mechanisms, interfacial stability and structural evolution during cycling remain inadequately addressed. In this review, we systematically trace the evolution of halide CAMs from liquid electrolyte systems to ASSLBs, focusing on recent breakthroughs in single-phase interface engineering and multi-electron reaction mechanisms. We highlight how elemental and structural units govern ionic/electronic transport pathways and reversibility, while critically evaluating the dilemma between energy density and efficiency in the multi-electron reaction. Looking forward, a roadmap for next-generation halide CAM development is outlined, encompassing high-throughput material screening, controllable design of integrated all-in-one architectures and strategies to overcome voltage hysteresis and conversion kinetics limitations. By bridging fundamental insights with practical engineering, this review aims to provide actionable guidance for realizing high-energy, cost-effective ASSLBs.

## Teaser

This review highlights the intrinsic advantages of multi-electron reactions and single-phase interfaces in halide cathodes, enabling higher energy density, efficient charge transport and simpler ASSLB designs.

## INTRODUCTION

The commercialization of lithium-ion batteries (LIBs) has driven transformative progress in portable electronics, electric transportation and renewable energy storage. In commercial LIBs, cathode active materials (CAMs) have been primarily dominated by transition metal oxides, including layered oxides (e.g. LiCoO_2_ and LiNi_x_Co_y_Mn_1-x-y_O_2_), polyanion oxides [e.g. LiFePO_4_ (LFP)] and spinel oxides (e.g. LiMn_2_O_4_), based on the intercalation mechanism. Despite widespread employment, their limited specific capacity (<250 mAh g^−^^1^) constrains their energy densities [1]. Additionally, existing oxide CAMs struggle to balance energy density, cycle life and safety, making it difficult to meet the growing demands of next-generation energy storage. These CAMs operate by facilitating reversible lithium ion (Li^+^) (de)intercalation within a restricted electrochemical window to maintain structural integrity and ensure long-term cyclability. Nevertheless, aggressive high-voltage charging or deep delithiation states induce severe lattice strain, irreversible phase transitions and progressive chemo-mechanical degradation. These degradation mechanisms limit Li utilization and accessible capacity, thereby compromising the overall energy output [2]. Consequently, exploring new CAMs beyond conventional oxide CAMs has become increasingly urgent.

Metal halides, dating back to the 1960s, have garnered significant interest as promising CAM candidates, due to their high theoretical energy densities and economic advantages. A representative case is the economical and environmentally friendly FeF_3_, which theoretically reacts with three Li^+^ and delivers a high capacity of 712 mAh g^−^^1^ at an average potential of ∼2.7 V, enabling a high theoretical energy density of 1950 Wh kg^−^^1^ [3]. Recently, FeCl_3_, as a high stability (83% capacity remaining after 1000 cycles) and cost-effective (cost 2% of the LFP) CAM, has been proven to be a promising candidate in all-solid-state lithium batteries (ASSLBs) [4]. The successful application of chloride CAMs in ASSLBs has solved the long-standing problem of dissolution in liquid electrolytes, enabling the development of more halide CAMs with unique properties. Nevertheless, the transition from liquid LIBs to ASSLBs introduces new design challenges. Conventional electrodes, inherited from liquid LIBs, rely on effective triple-phase contact among the CAMs, ion conductors (electrolyte) and electron conductors (carbon additives) to facilitate charge transfer. While liquid electrolytes ensure seamless interfacial wetting, the solid–solid interfaces in ASSLBs suffer from poor contact, severely limiting these triple-phase interfaces [5]. Consequently, ASSLBs require high fractions of inactive solid-state electrolytes (SSEs) and/or conductive carbon to maintain a charge transfer network, which inevitably compromises both the gravimetric and volumetric energy densities.

In this context, developing lithiated halide CAMs, such as Li_3_TiCl_6_ [6], Li_3_VCl_6_ [7] and Li_1.3_Fe_1.2_Cl_4_ [8], offers a promising alternative. Through open framework structure and rich elemental-structural regulatory methods, halide CAMs combine high ionic (σ_Li_, up to 10^−^^3^ S cm^−^^1^) and electronic conductivity (σ_e_, up to 10^−^^5^ S cm^−^^1^), enabling simplified architectures with low-additive content (<10 wt.%), thereby representing a critical pathway toward high-energy-density and practical ASSLBs [9]. Notably, despite the considerable promise outlined above, research on halide CAMs for ASSLBs remains in its early stages, and a dedicated review that comprehensively elucidates their charge-transfer kinetics, interfacial challenges, structural evolution and strategic design principles is urgently required.

This review aims to outline the development background, key challenges and recent advancements of halide CAMs for ASSLBs. We begin by tracing the history and classification of halide CAMs, highlighting their distinct advantages over conventional oxide CAMs. Despite significant progress, critical challenges remain, particularly in achieving effective interfacial regulation and developing a fundamental understanding of the underlying reaction mechanisms. Building on this, we focus on recent advances in structural design and interface engineering of halide CAMs, and propose an innovative strategy for constructing single-phase interfaces. This is accomplished through three complementary approaches: improving Li^+^ conductivity via structural tuning, enhancing σ_e_ based on energy band theory and boosting interfacial compatibility through targeted optimization. Moving beyond state-of-the-art descriptions, a paradigm shift in reaction mechanisms is elucidated, from a conventional single-electron intercalation mechanism to a multi-electron transfer mechanism, as a key pathway for enhancing energy density. Finally, perspectives on promising future research directions to solve the remaining challenges in materials design, in-depth mechanism analysis and device integration are presented. These include artificial intelligence (AI)-driven high-throughput screening for rapid material discovery, advanced characterization and simulation to unravel complex intercalation-conversion mechanisms, the development of high-voltage and integrated halide CAMs with optimized interfacial compatibility and scalable electrode fabrication processes toward high-energy-density ASSLB devices. Collectively, these avenues offer significant potential for advancing halide CAM research toward practical ASSLB applications.

## HALIDE CAMS: FROM LIBS TO ASSLBS

Halides (including non-lithiated MX_n_ and lithiated Li_a_MX_b_ compounds, where M represents transition metal ions and X denotes halogen ligands) can be traced to their initial application in LIBs, which attracted attention due to their high capacity, positioning them as promising alternatives to conventional intercalation-type oxide CAMs. While the high solubility of halides (excluding fluorides) in liquid electrolytes poses a significant challenge to their widespread use, this limitation has been circumvented by the advent of SSEs. The development of highly ion-conductive SSEs has paved the way for ASSLBs, repositioning halide compounds as highly promising CAM candidates. Their appeal lies in a unique combination of properties: high σ_Li_, a soft mechanical nature that ensures good interfacial compatibility with SSEs, and the potential for high capacity through multi-electron reactions. This section presents a concise overview of the history and classification of halide CAMs.

### The development history of halide CAMs

**Figure 1.**
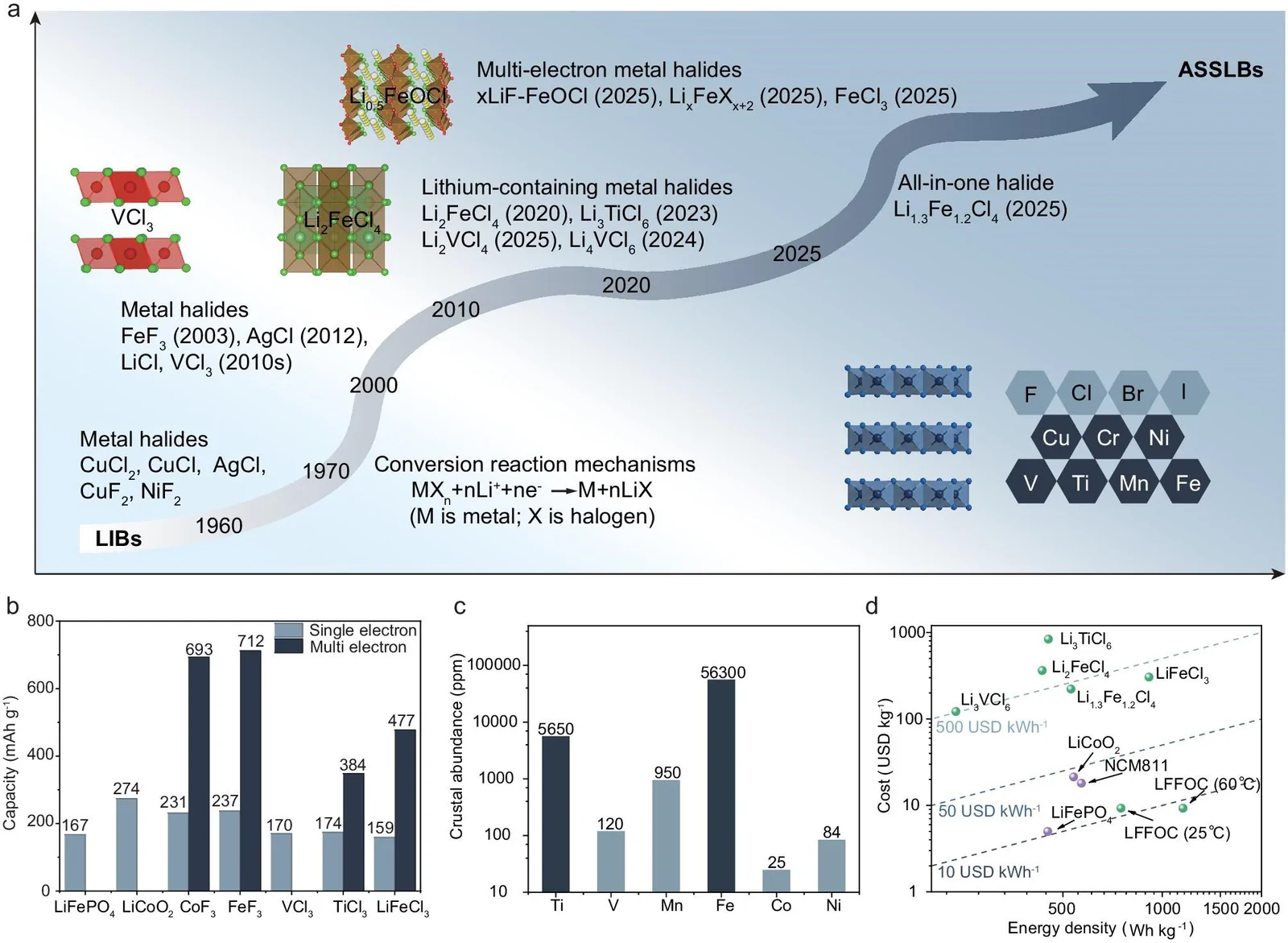
(a) The development history of halide CAMs. (b) The capacity of oxide and halide CAMs during single-electron transfer and multi-electron transfer. (c) The abundance of transition metal elements used in oxide and halide CAMs in the earth’s crust. (d) The energy density and cost of various CAMs. The energy density at the cathode level (Wh kg^−^^1^) in the references is calculated by integrating the capacity–voltage curves. To guarantee that the energy density values are not affected by the anode materials, the voltages corresponding to different anodes are uniformly converted to those corresponding to Li metal anodes.

As shown in Fig. 1a, the genesis of halide CAMs can be traced to the 1960s, when military applications created urgent demands for high-energy-density storage systems [10]. During this period, intercalation-based oxide CAMs such as LiCoO_2_ remained immature, leaving a critical gap in high-performance CAMs. Halide CAMs emerged as promising candidates due to their conversion reaction mechanisms, which offered high specific capacities, coupled with moderate operating voltage plateaus and the inherent advantages of abundant raw material availability and cost-effectiveness. Research during this period focused predominantly on metal fluoride and chloride materials. F-based CAMs, such as CoF_2_, NiF_2_ and CuF_2_, demonstrated impressive theoretical energy densities owing to the high voltage and multi-electron transfer reaction [11,12]. However, these materials suffered from low active material utilization, poor reversibility and solubility in liquid electrolytes [13]. These challenges have proven recalcitrant to mitigation and ultimately led to a gradual decline in research interest. Concurrently, Cl-based CAMs, such as CuCl_2_ and AgCl, attracted attention for their low-cost advantages [14,15]. Nevertheless, their significant solubility in liquid electrolytes induced severe self-discharge phenomena, which also constrained their practical deployment in liquid LIBs.

Following the commercialization of intercalation-type CAMs in the 1990s, which offered superior operating voltages, exceptional reversibility and favorable compatibility in liquid LIBs [16], research into halide CAMs progressively diminished. Notwithstanding this general trend, FeF_3_ continued to attract sustained scholarly attention as a low-cost and high-capacity CAM [17,18]. Subsequent investigations employing advanced *in situ* characterization techniques elucidated the conversion reaction mechanisms of FeF_3_/FeF_2_ CAMs, establishing crucial theoretical foundations for future improvements in conversion-type CAM design [19–21]. Despite these mechanistic insights, the practical viability of FeF_3_ remained compromised by its intrinsically low σ_e_ and pronounced voltage hysteresis, precluding widespread commercial adoption [22,23].

In recent years, the maturation of solvent-free ASSLBs has fundamentally resolved the persistent challenge of dissolution that had historically plagued halide CAMs. Traditional halide CAMs, including FeF_3_ [24], VCl_3_ [25] and FeCl_3_ [4], have demonstrated remarkable energy densities and exceptional cycling stabilities within ASSLB configurations. Furthermore, the conceptual innovation of lithiated halide CAMs represented by Li_a_MCl_b_ formulations, such as Li_3_TiCl_6_ [6], Li_3_VCl_6_ [7] and Li_x_FeCl_2+x_ [26], has introduced intrinsic σ_Li_ within halide frameworks. These lithiated halide CAMs have emerged as focal points of contemporary research, offering promising pathways to simultaneously enhance the energy density and rate capability of ASSLBs.

After >60 years of cyclical development, halide CAMs have secured renewed application prospects in ASSLBs, aligning precisely with the dual strategic objectives of contemporary energy storage research: maximizing energy density and power density while minimizing costs. Regarding energy and power metrics, halide CAMs possess relatively high molar masses that generally render single-electron theoretical capacities inferior to those of oxide CAMs. However, the multi-electron transfer characteristics exhibited by halides such as FeF_2_ [27], TiCl_3_ [28], FeCl_3_ [29] and LiFeCl_3_ [26] enable practical specific capacities exceeding 400 mAh g^−^^1^, translating to energy densities surpassing 800 Wh kg^−^^1^, values substantially exceeding commercial oxide CAMs (Fig. 1b). Moreover, advances in modifying both ionic and electronic conductivities of halide CAMs have enabled CAM loadings to approach 90 wt.% in composite cathodes. Emerging all-in-one architectures go a step further, making possible CAMs that are entirely electrochemically active [8].

From an economic perspective, the constituent elements of halide CAMs demonstrate favorable crustal abundances, such as Fe (56 300 ppm) and Ti (5650 ppm), affording substantial raw material cost advantages compared to Co/Ni-based oxides (Fig. 1c). Cost-optimized halide CAM formulations such as 0.5LiF+FeOCl (LFFOC) achieve material costs below 10 USD kg^−^^1^, even undercutting LFP while delivering superior energy density [30] (Fig. 1d). This exceptional cost performance distinguishes halide CAMs from other high-energy-density CAMs that typically suffer from expensive material costs. Coupled with the high σ_Li_ of halide frameworks, halide CAMs exhibit an attractive balance between cost and energy density, positioning them as promising candidates for cost-effective and high-performance ASSLBs.

### Categorization of halide CAMs

Generally, halide CAMs are broadly classified into two categories: non-lithiated halide CAMs and lithiated halide CAMs, each offering distinct structural and electrochemical characteristics.

#### Non-lithiated halide CAMs

Binary transition metal halides (MX_n_), such as VCl_3_, FeCl_3_ and FeF_3_, have gained attention as promising CAMs for ASSLBs. Sourced from industrial raw materials, these compounds offer favorable cost-effectiveness, paving the way for scalable applications. Despite the high solubility of such halide CAMs in liquid electrolytes previously limiting their application in conventional LIBs, the resurgence of SSEs has revitalized their potential. The pairing of halide CAMs with halide/sulfide SSEs ensures strong chemical and structural compatibility, resulting in excellent deformability and enhanced interfacial stability in ASSLBs. These favorable properties contribute to prolonged cycle life [28]. Typically, V-based halide series VX_3_ (X = Cl, Br and I) exhibits a layered structure comparable to classical intercalation hosts like LiCoO_2_. During Li^+^ intercalation/deintercalation, VX_3_ undergoes reversible phase transitions, though the final discharged structure depends on the halide anion size. For instance, VI_3_ retains an O1-type layered arrangement upon full lithiation, whereas VCl_3_ and VBr_3_ transform into an O3-type structure, a difference attributed to the ionic radii (Cl^−^ < Br^−^ < I^−^) [31]. The working voltage is strongly affected by the halide anion, where VCl_3_ delivers the highest redox potential (∼3 V vs Li^+^/Li) due to the electronegative Cl^−^ and pronounced Cl 3*p* and V 3*d* orbital hybridization. Given the advantages, VCl_3_ demonstrates potential utilization in ASSLBs: Li|VCl_3_ ASSLBs coupled with an Li_3_InCl_6_ SSE demonstrated stable cycling over 200 cycles with 85% capacity retention at 6C [25]. Similarly, FeCl_3_ offers a cost-effective alternative, with a material cost ∼1% that of LiCoO_2_. Through the Fe^3+^/Fe^2+^ redox couple, FeCl_3_ operates at two stable voltage plateaus between 3.5 and 3.8 V versus Li^+^/Li, delivering a specific capacity of 159 mAh g^−^^1^ and an energy density of 558 Wh kg^−^^1^ based on CAM mass [4].

#### Lithiated halide CAMs

In the last section, the non-lithiated halide CAMs have been summarized to build ASSLBs thanks to their chemical/electrochemical compatibility with SSEs. However, these halide CAMs are neither ionic nor electronic conductors. As a result, substantial quantities of ionic and electronic conductive additives are required during cathode fabrication to establish effective transport pathways for both ions and electrons. The introduction of these inactive components, combined with the difficulty of constructing a high-efficiency triple-phase interface via solid–solid contacts, poses a significant constraint on enhancing the energy density of the resulting ASSLBs. Thus, the lithiated halide CAMs (Li_a_MX_b_) are developed by incorporating Li into the halide framework, yielding CAMs that retain redox activity while gaining enhanced ionic transport. Among the various lithiated halide CAMs, the V-based halides stand out as a particularly promising class, owing to their high structural stability and elevated operating voltage, which collectively endow these CAMs with exceptional electrochemical reversibility and high energy density. For instance, Li_3_VCl_6_ functions as a typical halide CAM, exhibiting decent Li⁺ conductivity (7.5 × 10^−^^5^ S cm^−^^1^) and redox activity, which delivers a reversible capacity of ∼80 mAh g^−^^1^ at 3 V versus Li^+^/Li. When paired with LFP, it enables a high capacity of 217.1 mAh g^−^^1^_LFP_ and boosts energy density by ∼50% compared to the systems using inactive catholytes [7]. Additionally, Li_2_VCl_4_ is another potential CAM candidate, offering an σ_Li_ of 1–3 × 10^−^^5^ S cm^−^^1^ and a theoretical capacity of 129.7 mAh g^−^^1^ [32]. The exploration of halide CAMs also extends to lithiated Fe-based compounds. Li_2_FeCl_4_, initially studied as a cost-effective ionic conductor with a Li^+^ conductivity of 2.1 × 10^−^^5^ S cm^−^^1^, has been shown to exhibit reversible redox activity, delivering a capacity of 126 mAh g^−^^1^ at ∼3.6 V [33]. When integrated into ASSLBs, it retains 86% of its initial capacity after 6000 cycles at 2C [34]. Moreover, by activating the conversion reaction of Fe^2+^/Fe^0^, a three-electron transfer mechanism can be reached, yielding a high energy density of 912 Wh kg^−^^1^ with an acceptable cost-effectiveness for LiFeCl_3_ [26].

Overall, halide CAMs offer competitive operating potentials and capacities comparable to conventional oxide CAMs. In addition, their structural compatibility with halide SSEs and their tunable redox properties position as a promising pathway toward the development of homogeneous, high-performance cathodes for next-generation ASSLBs.

## HALIDE CAMS: FROM MULTI-PHASE TO SINGLE-PHASE INTERFACE

Conventional oxide CAMs are inherently poor ionic (10^−^^9^ to 10^−^^6^ S cm^−^^1^) and electronic conductors. Consequently, they require substantial amounts of catholytes (15–30 wt.%) and conductive agents to establish ionic and electronic transport pathways, enabling electrochemical reactions to occur at triple-phase boundaries where the ionic conductor, electronic conductor and CAM intersect [26]. However, in solid–solid contact systems, constructing and maintaining efficient triple-phase interfaces is challenging. CAMs are prone to losing contact with either the ionic or electronic conductor during cycling, leading to underutilization of the CAMs. Moreover, the resulting ion and electron transport pathways are often tortuous and lengthy, limiting the achievable capacity, energy density and rate capability of ASSLBs.

These challenges are expected to be addressed through the development of halide CAMs. By tailoring their crystal and band structures, both σ_Li_/σ_e_ can be significantly enhanced. This enables a transition from a complex triple-phase interface to a simplified dual-phase or even single-phase interface, ultimately allowing for the realization of all-in-one CAMs. Such an approach shortens ion and electron transport paths, simplifies interfacial architecture and facilitates self-sufficient transport networks within the cathode. This section comprehensively reviews a key strategy for boosting energy density via halide CAM innovation: the transition from multi-phase to single-phase interfaces. Building on this foundation, recent advances in boosting σ_Li_/σ_e_ of halide CAMs are underscored, followed by a discussion of interface optimization strategies, particularly tailoring the mechanical properties of halide CAMs and modulating CAM/SSE interactions.

### Ion/electron transport mechanism and enhancement strategies

As previously discussed, halide CAMs with mixed ionic-electronic conductivity provide several distinct advantages: maximized CAM utilization (theoretically up to 100%), minimized transport pathway tortuosity and elimination of CAM/SSE interface degradation. Thus, achieving such mixed conductivity is central to high-performance CAM design, necessitating a detailed understanding and precise modulation of σ_Li_/σ_e_ in halide CAMs. In this section, we will explore the origins of mixed conductivity through the lenses of solid-state ionic and energy band theory, followed by a review of recent advances in halide CAMs with engineered conductive properties.

#### Modulation of ionic conductivity

In composite cathodes for ASSLBs, the absence of liquid electrolyte infiltration necessitates the addition of 15–30 wt.% SSEs to construct ion transport networks, thereby limiting improvements in energy density of ASSLBs. For halide CAMs, structural similarity with halide SSEs, enables more facile achievement of high σ_Li_ than oxide CAMs, allowing the construction of catholyte-free composite cathodes. Similar to halide SSEs, distinct strategies are required for enhancing σ_Li_ in crystalline and amorphous halide CAMs.

##### Crystalline halide frameworks.

The σ_Li_ of crystalline halide CAMs is governed by their anionic framework topology, which dictates the size of diffusion bottlenecks, the geometry of Li⁺ migration pathways and the associated activation energy. For instance, most reported chloride materials crystallize in a close-packed anion sublattice, such as hexagonal (hcp) or cubic close packing (ccp) [35]. According to relevant research on halide SSEs, the anionic framework of crystalline halide materials is mainly influenced by ion migration pathways and the strength of anion/cation interactions. Inspired by these understandings, defect engineering and ion potential engineering, as the two main methods, are also applicable to improve the ion transport performance of crystalline halide CAMs.

**Figure 2.**
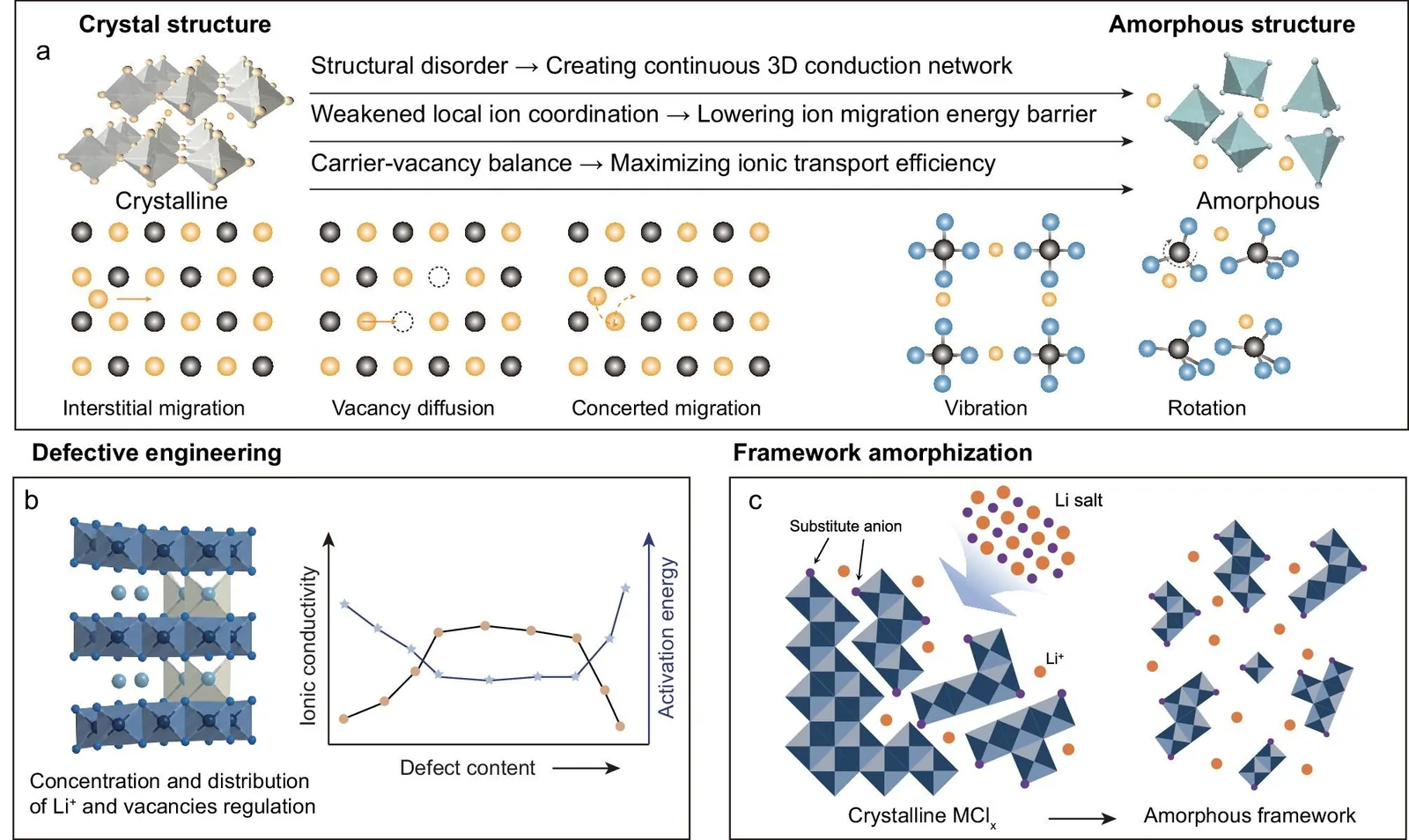
Strategies for enhancing σ_Li_ in halide CAMs. (a) The ionic transport models for the crystal and amorphous structures in halide CAMs, and the advantages of the amorphous structure. Variation trends of σ_Li_ and activation energy in (b) crystal defect engineering and (c) amorphous structure regulation.

To date, the ion migration mechanisms of crystalline halides mainly include vacancy-mediated hopping, interstitial diffusion and concerted migration (Fig. 2a) [36]. In crystalline halide materials, vacancy-mediated hopping is the main form of ion transport. Consequently, defect chemistry plays a decisive role in Li⁺ mobility. The regulation of Li⁺ mobility by defect engineering is mainly reflected in two aspects: vacancy concentration regulation and Li^+^ concentration regulation (Fig. 2b). As one of the most common methods, optimizing the occupancy of sites can be achieved by simply adjusting the stoichiometric ratio of raw materials [37,38]. Specifically, by varying the LiCl/ScCl_3_ molar ratio in Li_x_ScCl_3+x_, the occupation of Sc^3+^ and vacancies/Li^+^ can be precisely tuned to minimize the Sc-blocking effect in the vacancy-rich layers, resulting in a σ_Li_ of 3.02 × 10^−^^3^ S cm^−^^1^ and a low activation energy of 0.26 eV for Li_3_ScCl_6_ [39]. For another method, chemical substitution, especially aliovalent substitution, has emerged as another powerful strategy to simultaneously optimize the concentration of vacancy/Li^+^ [40,41]. For instance, in ccp-type halides, substituting Lu^3+^ with Zr^4+^ in Li_3-x_Lu_1-x_Zr_x_Cl_6_ reduces Li^+^ content while increasing vacancy concentration [42]. At 50% substitution, an optimal balance between Li⁺ and vacancies is achieved, yielding a maximal σ_Li_ of 1.50 × 10^−^^3^ S cm^−^^1^ and a minimal activation energy of 0.285 eV. Besides, aliovalent Fe^3+^ substitution into hcp-type Li_2_ZrCl_6_ simultaneously modulates the vacancy/Li^+^ concentration and induces lattice microstrain. Consequently, the Li^+^ conductivity is enhanced to ∼1 × 10^−^^3^ S cm^−^^1^ for Li_2.25_Zr_0.75_Fe_0.25_Cl_6_, accompanied by a significantly reduced activation energy of 0.346 eV [43].

Similar approaches have also been used to enhance the σ_Li_ of halide CAMs. For Fe-based halide CAM Li_2_FeCl_4_, which has been investigated as an SSE in the 1980s [44,45], is a typical halide CAM with a high intrinsic σ_Li_ of 1–2 × 10^−^^5^ S cm^−^^1^ [34]. On this basis, LiFeCl_3_ synthesized by non-stoichiometric methods exhibits defect regulation over Li_2_FeCl_4_ structurally, resulting in a higher σ_Li_ of 3.6 × 10^−^^5^ S cm^−^^1^ [26]. Furthermore, by introducing aliovalent Fe^3+^ to further regulate defects, the σ_Li_ of Li_1.3_Fe_1.2_Cl_4_ can be further increased to 2.28 × 10^−^^4^ S cm^−^^1^. Ab initio molecular dynamics (AIMD) simulations have demonstrated the fast three-dimensional Li^+^ diffusion network in Li_1.3_Fe_1.2_Cl_4_, which is the origin of high σ_Li_ [8]. For another halide CAM Li_2-x_VCl_4_, a solid-solution series with tunable Li content, experiences lattice expansion with increasing Li concentration, consistent with a topotactic (de)intercalation mechanism between V^2+^ and V^3+^. The resulting σ_Li_ ranges 1–3 × 10^−^^5^ S cm^−^^1^, varying systematically with stoichiometry [32]. When the stoichiometric ratio further changes to Li_3_VCl_6_, more defect sites cause the crystal structure to transition from cubic to monoclinic phase, creating more interconnected ion transport channels, resulting in a higher ion conductivity of 7.5 × 10^−^^5^ S cm^−^^1^ [7]. These results demonstrate that stoichiometry regulation enables precise control over defect populations and Li-vacancy arrangements, thereby improving ion transport performance.

Beyond structural defect engineering strategies, the ionic potential regulation strategy based on cation polarizing power and lattice chemistry serves as a complementary rational design approach for enhancing ionic transport capabilities [46,47]. Regarding cation doping approaches, the incorporation of high-ionic-potential cations (e.g. Ta^5+^ and Nb^5+^) not only generates vacancies but also leverages their strong polarization effect to weaken Li–Cl interactions, yielding a series of SSEs with σ_Li_ exceeding 1 × 10^−^^3^ S cm^−^^1^ [48–50]. More notably, studies have demonstrated that Ta^5+^ and Nb^5+^ can activate reversible redox activity, contributing an additional capacity of 60–100 mAh g^−^^1^ in Na-based all-solid-state batteries [51]. This discovery inspires the design of halide CAMs with high σ_Li_ based on high-ionic-potential cations.

##### Amorphous halide frameworks.

In contrast to rigid anionic frameworks that confine Li⁺ migration within narrow channels, transport mechanisms involving anionic motion in amorphous halides rely on a more flexible, non-close-packed framework. This structure alleviates the energetic heterogeneity of Li^+^ sites, resulting in a flattened energy landscape that manifests as reduced activation energy barriers and consequently enhanced σ_Li_ (Fig. 2a). Advanced characterization techniques, including molecular dynamics simulations, pair distribution function analysis and X-ray absorption spectroscopy, have elucidated how local anion dynamics (vibration and rotation) facilitate Li⁺ migration. For example, in glassy LiTaCl_6_, large-amplitude vibrations of Cl^−^ anions within TaCl_6_ units enable rapid Li⁺ hopping through repeated breaking and reforming of Li–Cl bonds, analogous to a ‘monkey-bar’ mechanism [52]. Similarly, the ‘soft-cradle’ effect identified in the LiMXCl_4_ family (where M = Ta, Nb, Sb, Ti, Zr, Hf and Sn, and X = O, F and Cl), links Li⁺ hopping to the tilting of coordination polyhedra, indicating that local anion rotation can effectively lower migration barriers and promote long-range ionic transport [53].

Considering the thermodynamic instability and structural complexity of amorphous materials, the precise and controllable realization of amorphization and the regulation of ion transport pathways are crucial for developing amorphous halide materials (Fig. 2c). By introducing an aliovalent anion such as O^2^^−^, the close-packed anionic framework can be disrupted, forming amorphous local structures based on bridging O/Cl, and enabling rapid Li^+^ transport through corner-sharing O atoms and Cl-rich sites [54,55]. A series of halide SSEs have achieved high σ_Li_ at the 1 × 10^−^^3^ S cm^−^^1^ level by regulating oxygen source types [56], doping concentrations [57] and synthesis methods [58,59]. Notably, Fe-based materials can contribute additional capacity while inducing amorphization reactions. For example, 1.2LiOH + FeCl_3_ features an O/Cl-bridged freely rotating Fe_x_O_y_Cl_z_ framework, achieving a high σ_Li_ of 6.1 × 10^−^^3^ S cm^−^^1^ and a reversible capacity of 97.7 mAh g^−^^1^ [60]. Besides, by introducing Fe_2_O_3_ into Li_2_ZrCl_6_, an amorphous ion transport network based on Zr-O/Cl, Fe-O/Cl and Li-Cl can be formed, enhancing the σ_Li_ to 2.55 × 10^−^^3^ S cm^−^^1^ while achieving a reversible capacity of 163 mAh g^−^^1^ [61].

Furthermore, some low-dimensional halide/oxyhalide materials condensed by van der Waals forces have been demonstrated to undergo solid dissociation processes, thereby facilitating the formation of amorphous structures more easily [62]. Such van der Waals crystals are widely present in redox-active transition metal halides, such as VCl_3_, FeCl_3_, FeOCl, CrOCl, etc., providing a foundation for constructing amorphous halide CAMs. For example, 0.5LiF + FeOCl amorphous halide CAM enhances the σ_Li_ of Fe-based halide CAMs from 10^−^^5^ S cm^−^^1^ to 2.6 × 10^−^^4^ S cm^−^^1^ through the O/F-induced amorphization process, benefiting from the promotion of ion transport by the amorphous ion transport pathway [30].

Overall, enhancing the σ_Li_ of halide materials hinges on precise structural control to regulate ion transport mechanisms. In crystalline halides, defect chemistry serves to balance Li⁺ and vacancy concentrations while optimizing migration pathways. Conversely, amorphous halide frameworks provide customized local disordered coordination environments, lower activation energy barriers and promote the formation of three-dimensional ion transport networks. Ultimately, integrating defect-engineered crystalline units with dynamically disordered amorphous domains establishes a versatile material design paradigm. This approach enables halide CAMs that simultaneously achieve high ionic mobility and robust electrochemical performance, enabling cathode formulations to realize efficient transport with extremely low SSE content, paving the way toward high-energy-density ASSLBs.

#### Modulation of electronic conductivity

Halide materials are typically characterized by wide bandgaps, leading to intrinsic σ_e_ below 10^−^^6^ S cm^−^^1^. For halide SSEs, low σ_e_ can suppress interface side reactions and self-discharge. But for halide CAMs, poor σ_e_ severely limits electron transport and hinders the reaction kinetics. Therefore, achieving high σ_e_ is particularly critical, as it facilitates the formation of efficient conductive networks and promotes essential redox reactions [63]. Referencing modification strategies for low-conductivity CAMs such as LFP, carbon coating effectively enhances σ_e_. However, the conductive carbon and CAM constitute a non-interacting two-phase system, thereby limiting interfacial electron transport due to solid–solid contact constraints. Therefore, current approaches to constructing single-phase interfaces primarily involve enhancing the intrinsic σ_e_ of halide materials via band structure engineering strategies.

**Figure 3.**
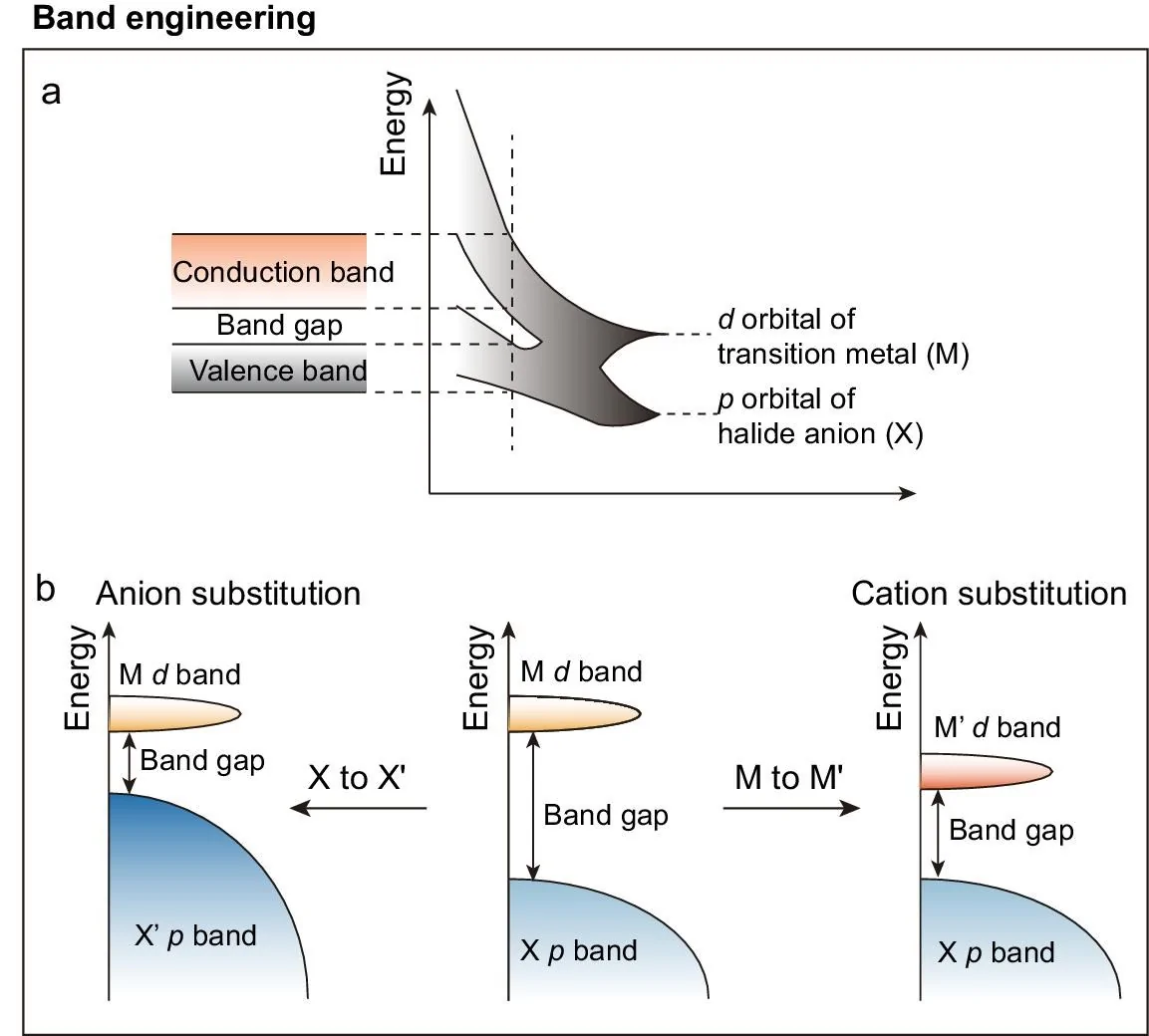
Strategies for enhancing σ_e_ in halide CAMs by band engineering. (a) The electronic band structure originates from the dispersion of atomic orbitals in a periodic lattice. (b) Band engineering strategies through anion and cation substitution.

To achieve precise band engineering, a fundamental understanding of the electronic structure of halide materials is essential (Fig. 3a). In halides, the valence and conduction bands arise from hybridization between transition metal *d* orbitals and halide *p* orbitals. Thus, introducing transition metal cations with partially filled *d* orbitals or raising the *p* orbital energy of the halide anions can create gap electronic states (Fig. 3b). This effect narrows the fundamental bandgap and increases the charge carrier concentration. For example, in nearly electronically insulating oxide LFP, the introduction of high-valence cations such as Ti^4+^, Nb^5+^ and Zr^4+^ forms Li_1-x_M_x_FePO_4_ solid solutions. Mixed valence states create acceptor levels near the valence band maximum (VBM), increasing hole/carrier concentration. The doped LFP exhibits an σ_e_ enhancement of ∼10^8^-fold, achieving minimal polarization even at ultrahigh current densities of 6000 mA g^−^^1^ (40C) [64]. Analogous modification strategies have been extended to halide CAMs. Through the introduction of Fe^3+^ into the Li_2_FeCl_4_ structure, Li_1.3_Fe_1.2_Cl_4_ establishes a mixed-valence Fe^3+^/Fe^2+^ framework, wherein the Fe^3+^/Fe^2+^ redox couples serve as electron hopping sites, thereby enhancing σ_e_ from 2.68 × 10^−^^9^ to 6.15 × 10^−^^5^ S cm^−^^1^. Leveraging the inherently high σ_Li_ of halide materials, this strategy enables the construction of single-phase all-in-one CAMs exhibiting excellent rate capability and cycling stability [8].

From the perspective of anions (O^2^^−^, S^2^^−^, Cl^−^, etc.), their *p* orbitals dominate the VBM position, and their electronegativity and orbital energy directly determine the bandgap width and redox potential. Anion substitution is also an effective means to increase the σ_e_ of CAMs. In halide materials, the high electronegativity of Cl^−^ results in a lower energy of VBM, which brings higher oxidation potential while significantly widening the bandgap. Therefore, substituting Cl^−^ with anions of lower electronegativity can obtain a smaller bandgap. In halide SSEs, theoretical studies predict that Li_3_InCl_0.33_Br_5.67_ can reduce the bandgap from 3.36 to 2.34 eV through Br^−^ substitution for Cl^−^ in Li_3_InCl_6_ [65]. Subsequent experimental studies have confirmed this understanding; the introduction of Br^−^ shifts the VBM upward in Li_3_InCl_6_, thereby reducing the bandgap [66]. However, even with Br-dominated *p* orbitals, the bandgap remains large (>2 eV), making it difficult to meet the requirements of CAM for high σ_e_ alone.

As another strategy, doping with S^2^^−^ and Se^2^^−^ anions may be a more effective means of band structure regulation. S^2^^−^ and Se^2^^−^ anions have higher *p*-band energy and actively narrow the bandgap by strengthening the hybridization between anion *p* orbitals and cation *d* orbitals [67]. For instance, S^2^^−^/Se^2^^−^ can be utilized to regulate the σ_e_ of CAM for designing two-phase or even single-phase composite cathodes in ASSLBs. As a representative material, Li_1.75_Ti_2_(Ge_0.25_P_0.75_S_3.8_Se_0.2_)_3_ (LTGPSSe) improves σ_Li_ through Ge doping and σ_e_ through Se^2^^−^ doping, achieving σ_e_ of 0.242 S cm^−^^1^ while maintaining σ_Li_ of 2.2 × 10^−^^4^ S cm^−^^1^. Homogeneous cathodes constructed from LTGPSSe can achieve a high capacity of 250 mAh g^−^^1^ and cycle life exceeding 20 000 cycles at room temperature (RT) [68]. Beyond sulfide CAMs, such anionic regulation strategies can be extended to halide systems, where the integration of sulfide species offers a promising avenue to overcome the severe σ_e_ limitations inherent to halide CAMs. 92Li_2_S@8LiFeS_2_ provides a possibility for introducing anionic bandgap regulation strategies into halide CAMs. Utilizing the reaction between Li_2_S and highly reactive MX_n_ (such as FeCl_3_ and VCl_3_) to construct LiFeS_2_/LiVS_2_ coatings with high σ_e_, thereby significantly improving the utilization of Li_2_S [69].

Based on the above approaches, introducing appropriate amounts of high-valence cations or S/Se anion doping to regulate the band structure in halide CAMs may become the preferred method for improving σ_e_ and constructing single-phase homogeneous halide CAMs. Nevertheless, the optimization of σ_Li_ and σ_e_ should be approached as an integrated engineering endeavor rather than isolated modifications, ensuring that neither ionic nor electronic transport becomes the rate-limiting bottleneck in single-phase architectures. However, achieving simultaneous regulation of both conductivities through a single strategy remains challenging, given the divergent structural requirements for ionic migration and electronic conduction. In this context, high-entropy design and amorphization strategies emerge as promising holistic solutions: the former enables concurrent tuning of multiple transport parameters through multi-element disorder, while the latter constructs isotropic percolation networks with reduced activation barriers for both ionic and electronic migration, thereby offering feasible routes toward systematic mixed-conductivity optimization.

### Improvement of interface compatibility

The interface compatibility within ASSLBs, particularly at the CAM–SSE interface, serves as a critical factor that significantly distinguishes ASSLBs from liquid LIBs and constrains their performance [70,71]. Compared to oxide CAMs, halide CAMs exhibit substantially improved interfacial compatibility attributable to their superior deformability and better-matched chemical potential with the SSE. However, current halide CAMs still struggle to meet the demands of commercial ASSLBs operating under low external pressure during a prolonged lifespan [72]. Improving mechanical contact efficiency and chemical compatibility represent effective strategies to address these challenges.

**Figure 4.**
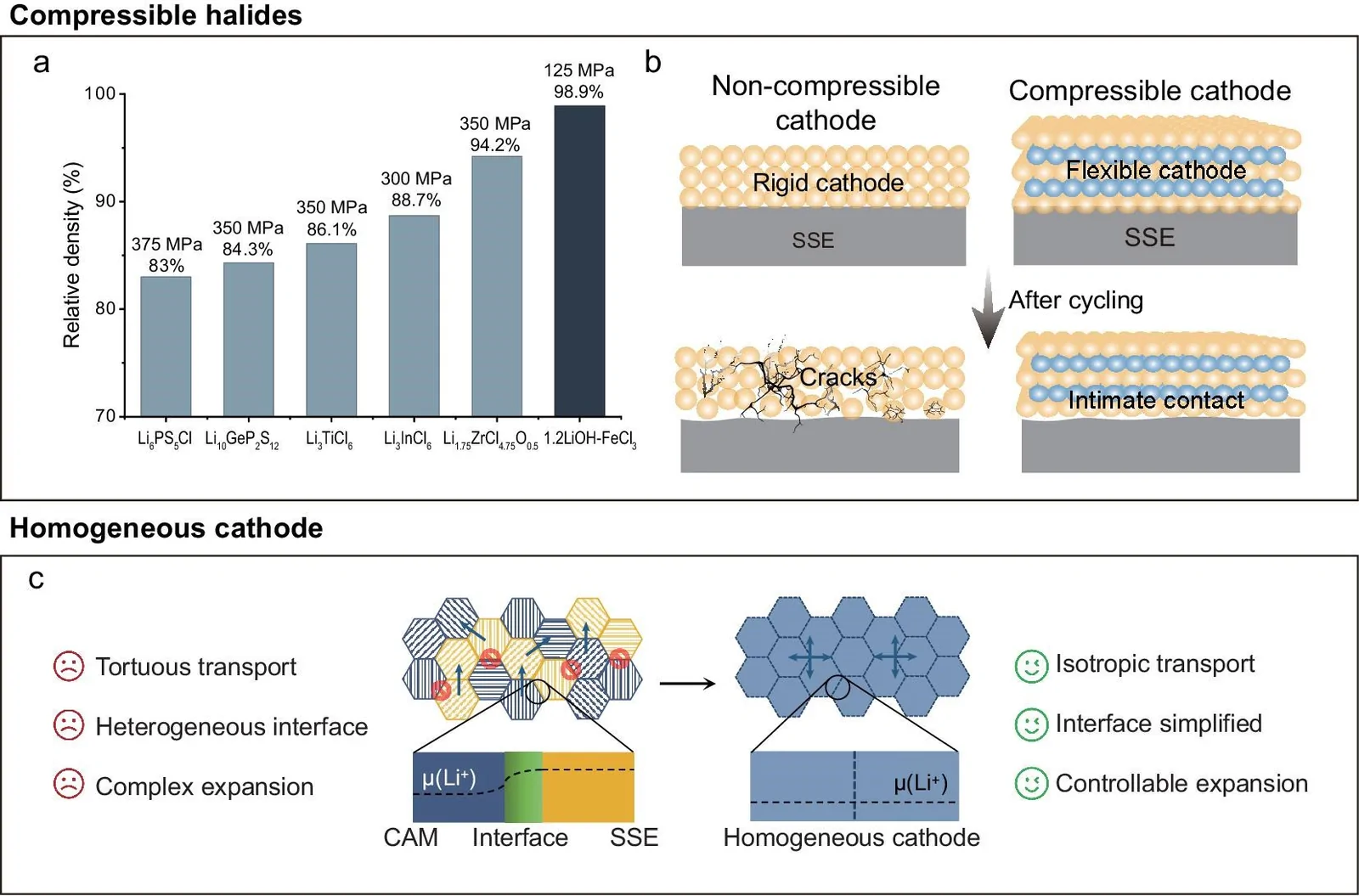
Interface compatibility of halide CAM/SSE. (a) Relative density of sulfide and halide CAM/SSE after compression. (b) Schematic illustration of the non-compressed cathode and the compressed cathode after cycling. (c) Comparison of ionic transport, interface contact and volume change between composite cathode and homogeneous cathode.

Whether favorable interparticle contact can be achieved to form stable and efficient ion transport pathways is currently one of the focal points for CAMs and SSEs in ASSLBs. Benefiting from the open structural framework of chloride materials and the high polarizability of Cl^−^, chloride SSEs and CAMs typically exhibit low Young’s modulus and high compressibility, enabling extremely low porosity under cold-pressing conditions (Fig. 4a) [73]. For example, Li_3_TiCl_6_ CAM achieved a relative density 86.1% under cold pressing at 360 MPa, which is even higher than partial halide and sulfide SSEs. This enabled a σ_Li_ of 1.04 × 10^−^^3^ S cm^−^^1^ at RT and the realization of catholyte-free ASSLBs with 95 wt.% CAM content [6]. Although a fairly high σ_Li_ has been achieved, a relative density of <90% still poses challenges to the long-term stability and energy density of ASSLBs. The amorphization method is one of the preferred approaches to further enhance the compressibility of halide materials. A series of amorphous halide SSEs, such as 1.25Li_3_N-TaCl_5_ (90.61% at 500 MPa) [48] and Li_1.75_ZrCl_4.75_O_0.5_ (94.2% at 300 MPa) [57], have achieved higher cold-pressed relative density and lower Young’s modulus. Similar methods can be extended to halide CAMs. For example, 1.2LiOH + FeCl_3_ halide CAM achieved a relative density of 98.97% under a pressure of merely 125 MPa through a highly mobile bridge O-Cl amorphous matrix. The flexible characteristic alleviates the volume change of CAM, enabling stable operation of ASSLBs under zero external pressure (Fig. 4b) [60].

Furthermore, constructing single-phase homogeneous cathodes represents a promising approach to suppressing contact loss induced by electrode material volume changes. By eliminating SSEs and conductive carbon from the composite cathode, heterogeneous interfacial contact issues can be alleviated. Simultaneously, the predictability of volume changes is ensured, thereby enabling the design of composite cathodes free from cracks and voids throughout the lifespan (Fig. 4c) [9]. Certain CAMs with mixed ionic/electronic conducting characteristics have demonstrated the feasibility of this design concept, as they exhibit extended cycling life [68,74].

In addition to mechanical compliance, the chemical stability of the CAM/SSE interface is equally critical. The chemical stability of halide CAMs primarily focuses on their reactivity with SSEs. Research on halide CAMs demonstrates excellent interfacial compatibility with halide SSEs [4,25]. Building upon this favorable intrinsic compatibility, anionic doping offers an effective interfacial engineering strategy to further stabilize the CAM/SSE boundary and regulate the redox kinetics of halide CAMs. Particularly, F-doping modification can be employed to weaken the metal–halogen bonds in halide CAMs through chemical coupling effects, thereby facilitating redox reactions. As demonstrated in the FeCl_3_/Li_2_ZrCl_5.6_F_0.4_ composite cathode, a highly stable LiF-rich interfacial layer is constructed while simultaneously promoting the reversible transition of FeCl_3_ [29]. Apart from pairing with halide SSEs, matching halide CAMs with sulfide SSEs possessing higher commercial maturity and superior σ_Li_ represents another potential scheme. Drawing on similar design strategies from halide SSEs, introducing appropriate amounts of O/F doping helps to enhance interfacial compatibility with sulfide electrolytes, thereby improving the coulombic efficiency of ASSLBs [75].

## HALIDE CAMS: FROM SINGLE-ELECTRON TO MULTI-ELECTRON

As noted in Table 1, certain halide CAMs exhibit promising fast Li^+^ and/or electron conducting behavior. However, their achievable energy densities are constrained by single-electron redox reactions. Recently, multi-electron halide CAMs, particularly Fe-based compounds such as FeF_3_, which deliver a theoretical capacity of 712 mAh g^−^^1^ based on three-electron transfer, have emerged as the most promising candidates for achieving high energy density in ASSLBs. The transition from single-electron to multi-electron reactions fundamentally amplifies the demands on electrode architecture. Multi-electron transfer, particularly the intercalation-conversion coupling, is accompanied by severe volume changes and structural reconstruction that inevitably induce mechanical stress and contact loss at heterogeneous solid–solid interfaces. In this context, the single-phase and all-in-one cathode architectures discussed in the section entitled ‘HALIDE CAMS: FROM MULTI-PHASE TO SINGLE-PHASE INTERFACE’ become particularly critical. In this section, we focus on the multi-electron transfer halide CAMs and their unique intercalation-conversion mechanism.

**Table 1.** Typical halide CAMs and their corresponding physicochemical properties.

CAM	Discharge plateaus/V (vs Li^+^/Li)	Capacity/ mAh g^−^^1^	Ionic conductivity/S cm^−^^1^	Electronic conductivity/S cm^−^^1^	Ref.
Li_3_TiCl_6_	3.13	90	1.04 × 10^−^^3^	3.32 × 10^−^^7^	[6]
TiCl_3_	2.65 and 2.39	170.6			[28]
Li_2_VCl_4_	2.8	129.7	(1.0–3.0) × 10^−^^5^		[32]
Li_3_VCl_6_	3	∼80	0.075 × 10^−^^3^	1.4 × 10^−^^7^	[7]
Li_3_VCl_6_/LFP		217.1			
VCl_3_	2.96 and 2.78	170			[25]
FeF_2_	2.63	∼600	∼10^−^^4^		[27]
FeCl_3_	3.65	159			[4]
		497			[29]
Li_2_FeCl_4_	3.7	130	∼10^−^^5^		[34]
Li_2_FeCl_4_	3.77	122.4	2.73 × 10^−^^5^	(1.8–3.6) × 10^−^^9^	[76]
Li_2_FeCl_4_	3.6	126	2.1 × 10^−^^5^		[33]
Li_1.3_Fe_1.2_Cl_4_	3.65	145	2.28 × 10^−^^4^	6.98 × 10^−^^5^	[8]
0.5LiF-FeOCl	∼1.56/2.52 and ∼3.6/3.76	586	0.26 × 10^−^^3^	∼10^−^^6^	[30]
Li_x_FeX_x+2_ (X = Cl and Br)	∼3.7 and ∼1.5	446	3.6 × 10^−^^5^ (LiFeCl_3_)	1.35 × 10^−^^7^ (LiFeCl_3_)	[26]

### Single-electron (de)intercalation mechanism

**Figure 5.**
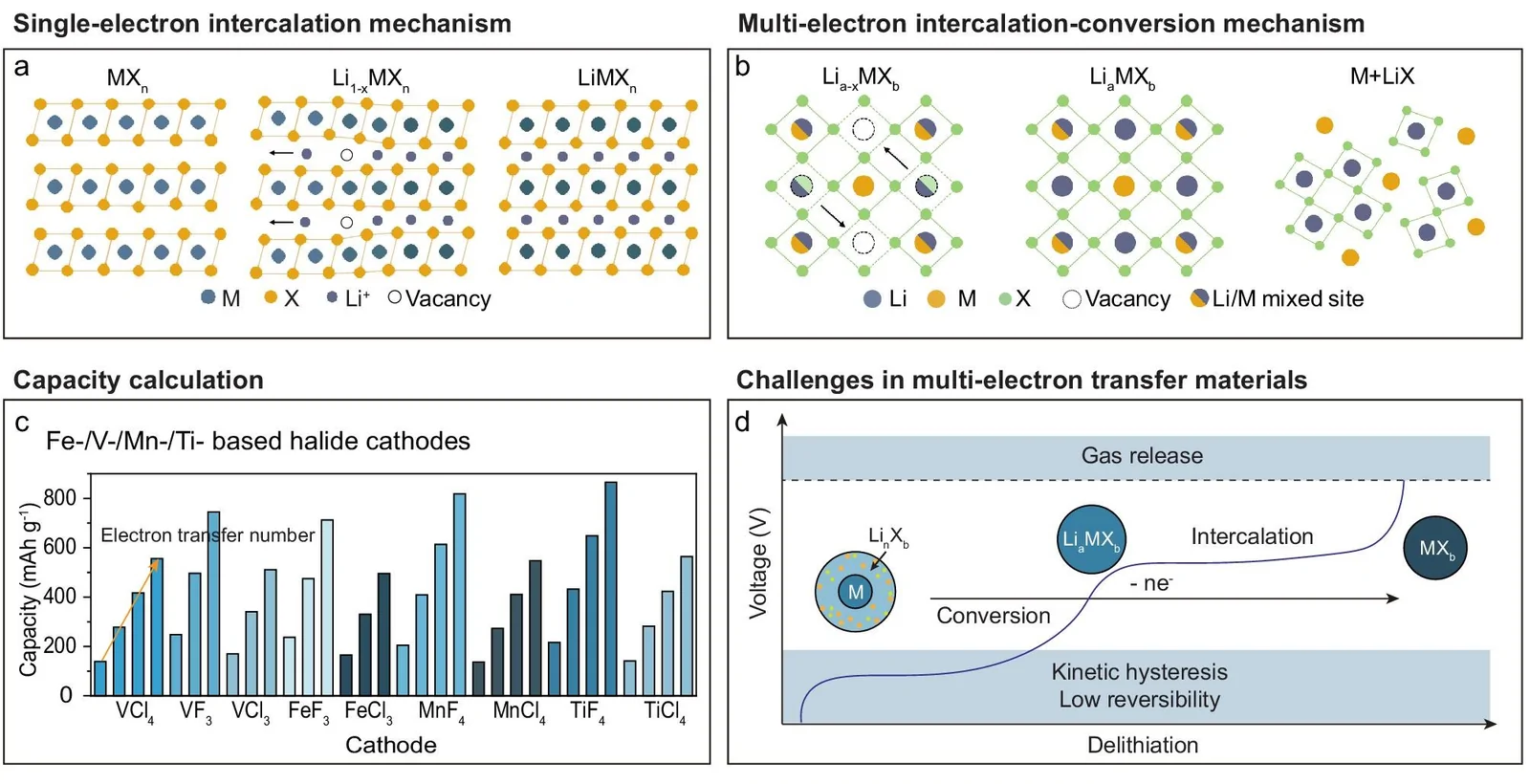
Halide CAMs based on (a) single-electron intercalation mechanism and (b) multi-electron intercalation-conversion mechanism. (c) Capacity predictions for several typical halide CAMs. (d) Challenges in multi-electron transfer CAMs.

Similar to the single-electron transfer mechanism observed in layered oxide CAMs, the electrochemical processes in halide CAMs are governed by Li^+^ intercalation and deintercalation. The underlying principle involves the two-dimensional reversible Li^+^ diffusion within the interlayers formed by transition metal oxide/halide frameworks. As depicted in Fig. 5a, during charging, Li^+^ is extracted while the transition metal is concurrently oxidized to maintain charge neutrality; the reverse occurs during discharge.

Although layered halides MCl_n_ (e.g. TiCl_3_ [28], VCl_3_ [25] and FeCl_3_ [4]) have structural similarities with LiCoO_2_, both rely on strong M–O or M–Cl ionic-covalent bonds and well-defined 2D diffusion channels. However, unlike LiCoO_2_, the M–Cl octahedral slabs in MCl_a_ are held together primarily by weak van der Waals forces. This structure allows Li⁺ to occupy two stable octahedral interlayer sites in a stepwise manner, resulting in a highly reversible reaction with minimal structural distortion and negligible phase transition. As a result, TiCl_3_ delivers exceptional cyclability, retaining 66.3% of its initial capacity after 900 cycles [28]. Also, FeCl_3_ exhibits better capacity retention, achieving 83% capacity remaining after 1000 cycles [4].

Layered characteristics are also found in lithiated halides of the type Li_a_MCl_b_ (e.g. Li_3_TiCl_6_, Li_3_VCl_6_ and Li_2_FeCl_4_), with the key distinction that additional lithium resides in the transition metal layer. Unlike MCl_n_, where MCl_6_ octahedral slabs are held together by van der Waals forces, all MCl_6_ octahedra in Li_a_MCl_b_ are connected via M–Cl ionic bonds, leading to substantially enhanced structural stability. Consequently, Li_3_TiCl_6_ achieves a capacity retention of 62.3% after 2500 cycles—∼2.5 times longer cycle life than TiCl_3_ [6]. In an even more striking case, Li_2_FeCl_4_ achieves a capacity retention of 86% after 6000 cycles, corresponding to a lifespan >6 times longer than that of FeCl_3_ [34]. Furthermore, the additional Li within the structural framework of Li_a_MCl_b_ forms intrinsic ion transport channels, enabling these materials to achieve a high intrinsic σ_Li_ of over 10^−^^4^ S cm^−^^1^ [6–8].

Nevertheless, halide CAMs are constrained by their single-electron transfer mechanisms, yielding specific capacities limited to ∼150 mAh g^−^^1^ and energy densities below 500 Wh kg^−^^1^, metrics that fall significantly short of competing with commercial oxide CAMs [4,7]. In contrast, state-of-the-art oxide CAMs have successfully harnessed multi-electron redox chemistry to surpass 250 mAh g^−^^1^ by activating both cationic and anionic redox centers. For instance, Ni-based layered oxides exploit the Ni^2+^/Ni^4+^ two-electron transfer couple [16,77], while Li-rich oxides achieve additional capacity through a reversible anion redox mechanism (O^2^^−^/O^n^^−^) [78,79]. Therefore, unlocking analogous multi-electron reactions in halide CAMs, beyond conventional single-electron transfer, emerges as a critical imperative for realizing a transformative leap in energy density.

### Multi-electron intercalation-conversion mechanism

Building on the limitations of single-electron transfer, multi-electron halide CAMs have attracted intense interest due to their potential for substantially higher energy density. Transition metal fluorides (MF_n_, M = Mn, Fe, Co, Ni, Cu, Cr, etc.), which operate based on multi-electron transfer conversion reactions, exhibit high specific capacities ranging from 500 to 800 mAh g^−^^1^, ∼3–7 times that of conventional commercial CAMs. Given their advantages of simple synthesis, low cost and abundant resources, they are regarded as promising next-generation high-performance CAMs [80]. Taking Fe-based fluorides as an example, their reaction mechanism involves a complex process combining two-step topological and two-step conversion reactions, accompanied by intricate structural topological changes during (de)lithiation (Fig. 5b) [19,21]. However, upon discharge, the accumulation of nanoscale conversion products (nano Fe and LiF) on the surface imposes a dual detrimental effect: (i) it masks the underlying active phases, shifting the reaction potential downward to ∼1.9 V versus Li⁺/Li for nano-Fe/LiF formation, and (ii) it physically blocks Li⁺ transport, as evidenced by a two-order-of-magnitude decrease in the Li⁺ diffusion coefficient and a sharp increase in polarization. These cascading effects cause severe voltage hysteresis. Notably, nearly all fluoride CAMs exhibit pronounced voltage hysteresis, with charge–discharge gaps exceeding 0.5 V, and, in some cases surpassing 1 V in CoF_2_, CrF_3_ and MnF_3_ systems. The substantial overpotential and severe voltage hysteresis directly culminate in severely limited voltage efficiencies: ∼65% for FeF_2_ and 62.7% for NiF_2_ [81,82]. Consequently, strategies that modulate redox reaction pathways, such as nanostructure engineering or high-entropy design, are considered effective approaches for mitigating voltage hysteresis in fluoride-based CAMs [20,83,84].

In light of the severe voltage hysteresis in fluorides, attention has turned to other, more structurally stable halide CAMs (MX_n_/Li_a_MX_b_, X = Cl, Br and I), which also offer excellent interfacial compatibility with SSEs. Li_3_TiCl_6_ operates via a pure intercalation-based two-electron process (Ti^2+^/Ti^3+^/Ti^4+^), where both electron transfers show high reversibility and fast kinetics due to its high intrinsic σ_Li_ [6]. Nevertheless, its practical application is hindered by the low voltage of the Ti^2+^/Ti^3+^ plateau and difficulties in stabilizing TiCl_3_ during synthesis. In contrast, LiFeCl_3_, FeCl_3_ and related compounds adopt a more aggressive three-electron intercalation-conversion coupling mechanism (Fe^0^/Fe^2+^/Fe^3+^). This approach, enabled by low-cost precursors, achieves capacities exceeding 400 mAh g^−^^1^ [26,29,30]. The reaction mechanism is similar to the intercalation-conversion coupling mechanism of FeF_3_, involving complex structural topological changes. However, this mechanism suffers from a substantial voltage disparity between its two redox plateaus, complicating anode matching and practical cell integration. Moreover, the conversion component exhibits sluggish kinetics, rapid capacity fade and poor CAM utilization, underscoring the inherent trade-off between electron transfer number and electrochemical reversibility. Considering this, hybridizing transition metal cations with distinct redox potentials or leveraging the high σ_Li_ of halide CAMs to composite with sulfur or oxide CAMs represents a promising strategy for optimizing the discharge characteristics of multi-electron transfer halide CAMs [60,74,85].

Overall, halides with multi-electron transfer reactions represent highly promising CAMs for future high-energy-density ASSLBs. In addition to the Fe- and Ti-based halide CAMs mentioned above, other transition metal cations capable of multi-electron conversion reactions, such as V, Cr and Mn, are also being explored as central cations in halide CAMs. As shown in Fig. 5c, TiF_4_ delivers a specific capacity as high as 800 mAh g^−^^1^. Notably, the application of halide CAMs is constrained by their limited electrochemical stability window (Fig. 5d). Charging beyond this window triggers distinct failure mechanisms: at high voltages (>4.2 V vs Li⁺/Li), excessive delithiation in halides may trigger detrimental structural degradation, including irreversible phase transitions and detrimental interfacial side reactions such as halogen gas evolution, causing serious safety hazards. To address detrimental interfacial side reactions and halogen gas evolution at high voltages, targeted stabilization strategies are required. (i) Protective coatings or *in situ* formed interphases, such as F-rich layers, can effectively arrest the continuous progression of parasitic reactions and impede halogen gas evolution [86]. (ii) Regulating the local chemical environment of transition metal centers to strengthen M–Cl bonding raises the energy barrier for Cl^−^ oxidation [87], thereby shifting the onset of halogen release to higher potentials and expanding the high-voltage operational limit. Conversely, at low voltages (<2 V vs Li⁺/Li), the formation of metallic species and lithiated halides tends to encapsulate the surface of CAMs, hindering ion transport and reducing reversibility. To mitigate severe structural reconstruction and irreversible capacity loss during multi-electron reactions in halide CAMs, several strategies merit consideration. Nanostructuring can shorten ionic diffusion pathways and increase active site exposure, thereby alleviating kinetic limitations and surface passivation during conversion. Reaction pathway modulation, analogous to the design evolution from FeF_3_ to FeOF, can transform destructive conversion reactions into topotactic transformations with minimal structural rearrangement. These material-level strategies collectively lay the groundwork for expanding the electrochemical stability window of multi-electron halide CAMs.

## SUMMARY AND OUTLOOK

**Figure 6.**
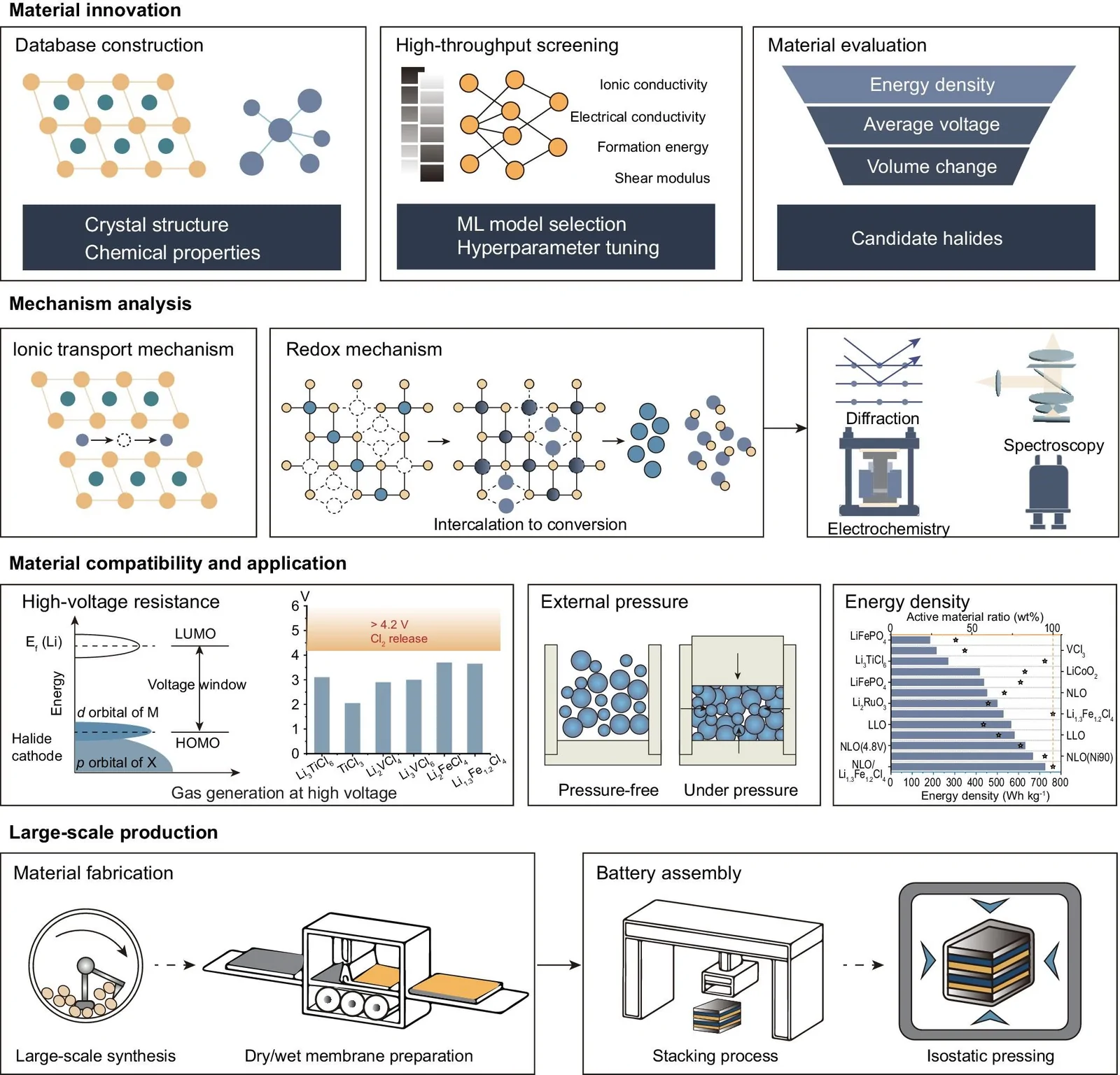
Future perspectives on halide CAMs. The energy density data are provided in Ref. [8].

In summary, recent progress in halide CAMs has been reviewed, along with the key challenges that still need to be addressed. As a relatively new candidate for ASSLBs, halide CAMs currently remain limited in material diversity, and many fundamental issues are not yet well understood. These include their compatibility with existing SSEs, interfacial stability, structural evolution and the relationship between these factors and electrochemical performance. In addition, the effects of charge compensation, phase-transition-induced changes in ion/electron transport pathways and the complex interplay between intercalation and conversion reactions require further investigation. For conversion-type halide CAMs, it is critical to ensure that newly formed phases remain electrochemically active and reversible during cycling. Improving conversion kinetics, reducing voltage hysteresis and narrowing the voltage gap between intercalation and conversion reactions are also essential for realizing practical multi-electron halide CAMs. Addressing these challenges will require new materials, advanced design strategies, theoretical models and a deeper understanding of this emerging material system. Future research may focus on high-throughput calculations for screening promising halide CAMs, the controllable design of integrated all-in-one halide CAMs with single-phase structures and high σ_Li_/σ_e_ and the development of high-capacity halide CAMs based on multi-electron transfer reactions. From the perspective of practical ASSLB applications, these directions provide useful guidance for future studies (Fig. 6).

### Halide innovation via AI-driven screening

The future advancement of halide CAMs hinges on the deep integration of materials science and AI. Through AI-driven screening, high-performance halide CAMs with superior σ_Li_/σ_e_ and favorable interfacial compatibility can be systematically identified from vast chemical and structural combinations, enabling integrated design and enhanced electrochemical performance. Specifically, based on known halide crystal structures, element substitution and defect engineering can be employed to generate thousands of derivative compositions.

Machine learning (ML) models are then applied to rapidly predict key properties such as σ_Li_, electrochemical stability and mechanical strength. This ‘predict-then-verify’ funnel-shaped screening strategy, which first uses fast ML models for initial filtering, followed by precise density functional theory or AIMD methods for validation, significantly accelerates material discovery, shifting the paradigm from traditional trial-and-error approaches to data-driven high-throughput development [36]. Moreover, AI-based modeling enables large-scale atomistic simulation of ion-transport behaviors, providing theoretical insights for designing novel amorphous structures, three-dimensional ion channels and integrated all-in-one CAMs, thereby accelerating the research and development of high-performance halide CAMs.

### Mechanism analysis via advanced characterization

A deep understanding of the ionic conduction mechanisms and intercalation-conversion reaction processes in CAMs, particularly their still-unclear failure behaviors, is crucial for future development. Advanced characterization techniques, such as *in situ* spectral characterization and isotope tracing, enable real-time ion intercalation/conversion processes, phase transformations and interfacial evolution, thereby revealing the microscopic origins of electrochemical performance. Specifically, for conversion-type halide CAMs, it is essential to clarify structural stability and reversibility during multi-electron transfer processes. For CAM/SSE interfaces in ASSLBs, elucidating the kinetic limiting factors of ion transport across interfaces is equally critical. Insights gained at the mechanistic level not only guide the structural optimization of materials, such as regulating lattice distortion or designing buffer layers, but also provide a theoretical foundation for enhancing ASSLB cycle life and rate capability. In the future, integrating multi-scale simulations with high spatiotemporal-resolution characterization will enable closed-loop mechanistic analysis from the atomic to the device level, advancing halide CAMs’ design toward rational regulation.

### Material compatibility and application

The practical application of halide CAMs depends on maintaining their high energy density. This can be achieved through three main strategies. (i) High-voltage halide CAMs should be designed to suppress gas evolution, such as Cl_2_ release at voltages above 4.2 V vs Li⁺/Li. To mitigate this, the redox-level tuning strategy is key. Partial substitution of Cl^−^ with more electronegative anions such as O^2^^−^ can tune the transition-metal redox level, enabling reversible metal redox below the gas-evolution threshold instead of irreversible Cl_2_ generation [88]. (ii) ‘All-in-one cathodes’ can reduce inactive components and improve electrode-level energy density by combining multi-functional CAMs. For example, leveraging reversible Fe^3+^/Fe^2+^ redox and fast ion/electron transport, Li_1.3_Fe_1.2_Cl_4_ delivers an energy density of 529.3 Wh kg^−^^1^ [8], while combining Li_2_FeCl_4_ with LiNi_0.92_Co_0.05_Mn_0.03_O_2_ achieves 766.5 Wh kg^−^^1^ at the electrode level [85]. (iii) Interfacial stress must be controlled because the large volume changes of CAMs during cycling can cause SSE cracking or contact loss [89]. Although external pressure can improve interfacial contact, it adds system weight and is impractical for real batteries. Therefore, stress-modulation strategies, such as flexible halide design and theoretical modeling, are essential for stable CAM/SSE interfaces without sacrificing energy density [90].

### Fabrication processes and industrial-scale control

Translating laboratory-scale halide CAMs into industrially viable ASSLBs requires integrated progress in material synthesis, electrode fabrication and battery assembly. (i) Scalable synthesis can be realized via mechanochemical or continuous solid-state processes with precise stoichiometry control. (ii) Electrode fabrication should support both wet and dry routes: wet processing may use non-polar solvents to avoid parasitic reactions, while dry processing enables solvent-free, densified electrodes, though uniform powder mixing and ultrathin dry-film formation remain challenges [91]. (iii) During battery assembly, accurate stacking of cathode, electrolyte and anode layers is essential for good interfacial contact, while isostatic pressing can remove interfacial voids and improve mechanical integrity for low-stack-pressure operation.

Moreover, the hygroscopic nature of halide materials requires strict moisture control, typically below −40°C dew point. Fortunately, recent advances in compositional modification and surface passivation have demonstrated viable routes to enhancing moisture tolerance [92,93]. Furthermore, the recently demonstrated water-mediated synthesis of halide SSEs such as Li_3_InCl_6_ offers a promising avenue for developing moisture-tolerant halide CAMs [94]. Collectively, coordinated advances in materials and manufacturing are needed to enable scalable halide-based ASSLBs.

## Supplementary Material

nwag438_Supplemental_File

## References

[1] Manthiram A, Cui ZH. Chemical factors controlling the behaviour of oxide cathodes in batteries. Nat Energy 2026; 11: 517–25. 10.1038/s41560-025-01963-x

[2] Liu T, Yu L, Liu J et al. Ultrastable cathodes enabled by compositional and structural dual-gradient design. Nat Energy 2024; 9: 1252–63. 10.1038/s41560-024-01605-8

[3] Li L, Meng F, Jin S. High-capacity lithium-ion battery conversion cathodes based on iron fluoride nanowires and insights into the conversion mechanism. Nano Lett 2012; 12: 6030–7. 10.1021/nl303630p 23106167

[4] Liu ZT, Liu J, Zhao SM et al. Low-cost iron trichloride cathode for all-solid-state lithium-ion batteries. Nat Sustain 2024; 7: 1492–500. 10.1038/s41893-024-01431-6

[5] Xiao Y, Wang Y, Bo S-H et al. Understanding interface stability in solid-state batteries. Nat Rev Mater 2020; 5: 105–26. 10.1038/s41578-019-0157-5

[6] Wang K, Gu Z, Xi Z et al. Li_3_TiCl_6_ as ionic conductive and compressible positive electrode active material for all-solid-state lithium-based batteries. Nat Commun 2023; 14: 1396. 10.1038/s41467-023-37122-7 36914653 PMC10011600

[7] Song Z, Dai Y, Wang T et al. An active halide catholyte boosts the extra capacity for all-solid-state batteries. Adv Mater 2024; 36: 2405277. 10.1002/adma.202405277 38877545

[8] Fu JM, Wang CH, Wang S et al. A cost-effective all-in-one halide material for all-solid-state batteries. Nature 2025; 643: 111–8. 10.1038/s41586-025-09153-1 40562942

[9] Xiong X, Jiang G, Li H et al. All-electrochem-active all solid state batteries. Energy Storage Mater 2025; 79: 104330. 10.1016/j.ensm.2025.104330

[10] Teng YT, Wei F, Yazami R. Synthesis of Ni_x_Co_1−x_F_2_ (x = 0, 0.25, 0.50, 0.75, 1.0) and application in lithium ion batteries. J Alloys Compd 2015; 653: 434–43. 10.1016/j.jallcom.2015.09.036

[11] Broussely M, Biensan P, Simon B. Lithium insertion into host materials: the key to success for Li ion batteries. Electrochim Acta 1999; 45: 3–22. 10.1016/S0013-4686(99)00189-9

[12] Vincent C . Lithium batteries: a 50-year perspective, 1959–2009. Solid State Ion 2000; 134: 159–67. 10.1016/S0167-2738(00)00723-2

[13] Li H, Balaya P, Maier J. Li-storage via heterogeneous reaction in selected binary metal fluorides and oxides. J Electrochem Soc 2004; 151: A1878–85. 10.1149/1.1801451

[14] Li T, Chen ZX, Cao YL et al. Transition-metal chlorides as conversion cathode materials for Li-ion batteries. Electrochim Acta 2012; 68: 202–5. 10.1016/j.electacta.2012.02.061

[15] Dai Y, Zhang S, Wen J et al. Metal chloride cathodes for next-generation rechargeable lithium batteries. iScience 2024; 27: 109557. 10.1016/j.isci.2024.109557 38623342 PMC11016933

[16] Manthiram A . A reflection on lithium-ion battery cathode chemistry. Nat Commun 2020; 11: 1550. 10.1038/s41467-020-15355-0 32214093 PMC7096394

[17] Arai H, Okada S, Sakurai Y et al. Cathode performance and voltage estimation of metal trihalides. J Power Sources 1997; 68: 716–9. 10.1016/S0378-7753(96)02580-3

[18] Conte DE, Pinna N. A review on the application of iron(III) fluorides as positive electrodes for secondary cells. Mater Renew Sustain Energy 2014; 3: 37. 10.1007/s40243-014-0037-2

[19] Karki K, Wu L, Ma Y et al. Revisiting conversion reaction mechanisms in lithium batteries: lithiation-driven topotactic transformation in FeF_2_. J Am Chem Soc 2018; 140: 17915–22. 10.1021/jacs.8b07740 30456949

[20] Xiao ABW, Lee HJ, Capone I et al. Understanding the conversion mechanism and performance of monodisperse FeF_2_ nanocrystal cathodes. Nat Mater 2020; 19: 644. 10.1038/s41563-020-0621-z 32094491

[21] Hua X, Eggeman AS, Castillo-Martínez E et al. Revisiting metal fluorides as lithium-ion battery cathodes. Nat Mater 2021; 20: 841–50. 10.1038/s41563-020-00893-1 33479526

[22] Fan XL, Hu EY, Ji X et al. High energy-density and reversibility of iron fluoride cathode enabled via an intercalation-extrusion reaction. Nat Commun 2018; 9: 2324. 10.1038/s41467-018-04476-2 29899467 PMC5998086

[23] Olbrich LF, Xiao AW, Pasta M. Conversion-type fluoride cathodes: current state of the art. Curr Opin Electrochem 2021; 30: 100779. 10.1016/j.coelec.2021.100779

[24] Wang X, Wang ZX, Chen LQ et al. High-capacity sulfide all-solid-state lithium battery with a conversion-type iron fluoride cathode. J Mater Chem A 2023; 11: 4142–54. 10.1039/D2TA09109G

[25] Liang J, Li X, Kim JT et al. Halide layer cathodes for compatible and fast-charged halides-based all-solid-state Li metal batteries. Angew Chem Int Ed 2023; 62: e202217081. 10.1002/anie.202217081 36697365

[26] Zhou X, Jiang M, Duan Y et al. Multi-electron transfer halide cathode materials based on intercalation-conversion reaction towards all-solid-state lithium batteries. Angew Chem Int Ed 2024; 64: e202416635. 10.1002/anie.202416635 39623746

[27] Shao B, Tan S, Huang Y et al. Enabling conversion-type iron fluoride cathode by halide-based solid electrolyte. Adv Funct Mater 2022; 32: 2206845. 10.1002/adfm.202206845

[28] Zhang RC, Gu ZQ, Sun SQ et al. Intercalation-type halide cathode material for all-solid-state lithium batteries. Nano Lett 2025; 25: 16404–10. 10.1021/acs.nanolett.5c04257 41187255

[29] Lin YC, Huang HQ, Xia YF et al. Interfacial coupling with halide solid electrolyte enables high-energy-density and reversible FeCl_3_ cathode for all-solid-state lithium batteries. Adv Energy Mater 2025; 15: e03269. 10.1002/aenm.202503269

[30] Duan Y, Hussain F, Liu H et al. Breaking the conductivity-capacity trade-off in MCl_6_ anionic framework: amorphous oxyhalide cathode materials enable ≈1100 wh kg^−^^1^ at cathode-level in all-solid-state lithium batteries. Adv Mater 2025; 37: e13544. 10.1002/adma.202513544 40872999

[31] Dubouis N, Marchandier T, Rousse G et al. Extending insertion electrochemistry to soluble layered halides with superconcentrated electrolytes. Nat Mater 2021; 20: 1545–50. 10.1038/s41563-021-01060-w 34326505

[32] Kasahara T, Song P, Honma I et al. Electrolyte-free cathode design for solid-state batteries demonstrated with bifunctional Li_2_VCl_4_. Batteries Supercaps 2024; 8: e202400520. 10.1002/batt.202400520

[33] Tanibata N, Kato M, Takimoto S et al. High formability and fast lithium diffusivity in metastable spinel chloride for rechargeable all-solid-state lithium-ion batteries. Adv Energy Sustain Res 2020; 1: 2000025. 10.1002/aesr.202000025

[34] Liu ZT, Zhang GX, Pepas J et al. Li_2_FeCl_4_ as a cost-effective and durable cathode for solid-state li-ion batteries. ACS Energy Lett 2024; 9: 5464–70. 10.1021/acsenergylett.4c02376 39539635 PMC11555680

[35] Ogbolu BO, Poudel TP, Dikella TNDD et al. Tailoring ion transport in Li_3-3y_Ho_1+y_Cl_6-x_Br_x_ via transition-metal free structural planes and charge carrier distribution. Adv Sci 2024; 12: 2409668. 10.1002/advs.202409668 PMC11831455 39690877

[36] Dutra ACC, Goldmann BA, Islam MS et al. Understanding solid-state battery electrolytes using atomistic modelling and machine learning. Nat Rev Mater 2025; 10: 566–83. 10.1038/s41578-025-00817-y

[37] Zhou L, Zuo T-T, Kwok CY et al. High areal capacity, long cycle life 4 V ceramic all-solid-state Li-ion batteries enabled by chloride solid electrolytes. Nat Energy 2022; 7: 83–93. 10.1038/s41560-021-00952-0

[38] Zhang S, Zhao F, Su H et al. Cubic iodide Li_x_YI_3+x_ superionic conductors through defect manipulation for all-solid-state Li batteries. Angew Chem Int Ed 2024; 63: e202316360. 10.1002/anie.202316360 38243690

[39] Liang JW, Li XN, Wang S et al. Site-occupation-tuned superionic Li_x_ScCl_3+x_ halide solid electrolytes for all-solid-state batteries. J Am Chem Soc 2020; 142: 7012–22. 10.1021/jacs.0c00134 32212650

[40] Park K-H, Kaup K, Assoud A et al. High-voltage superionic halide solid electrolytes for all-solid-state Li-ion batteries. ACS Energy Lett 2020; 5: 533–9. 10.1021/acsenergylett.9b02599

[41] Kim SY, Kaup K, Park K-H et al. Lithium ytterbium-based halide solid electrolytes for high voltage all-solid-state batteries. ACS Mater Lett 2021; 3: 930–8. 10.1021/acsmaterialslett.1c00142

[42] Wang CH, Wang S, Liu XD et al. New insights into aliovalent substituted halide solid electrolytes for cobalt-free all-solid state batteries. Energy Environ Sci 2023; 16: 5136–43. 10.1039/D3EE01119D

[43] Kwak H, Han D, Lyoo J et al. New cost-effective halide solid electrolytes for all-solid-state batteries: mechanochemically prepared Fe^3+^-substituted Li_2_ZrCl_6_. Adv Energy Mater 2021; 11: 2003190. 10.1002/aenm.202003190

[44] Lutz HD, Schmidt W, Haeuseler H. Chloride spinels: a new group of solid lithium electrolytes. J Phys Chem Solids 1981; 42: 287–9. 10.1016/0022-3697(81)90142-6

[45] Kanno R, Takeda Y, Takahashi A et al. Structure, ionic conductivity, and phase transformation in new polymorphs of the double chloride spinel, Li_2_FeCl_4_. J Solid State Chem 1988; 72: 363–75. 10.1016/0022-4596(88)90040-0

[46] Li XN, Kim JT, Luo J et al. Structural regulation of halide superionic conductors for all-solid-state lithium batteries. Nat Commun 2024; 15: 53. 10.1038/s41467-023-43886-9 38167381 PMC10761688

[47] Wang QD, Hu YS, Li H et al. Ionic potential for battery materials. Nat Rev Mater 2025; 10: 697–712. 10.1038/s41578-025-00822-1

[48] Hong B, Gao L, Li C et al. All-solid-state batteries designed for operation under extreme cold conditions. Nat Commun 2025; 16: 143. 10.1038/s41467-024-55154-5 39747842 PMC11696891

[49] Qian L, Tu SB, Wang Y et al. Near-saturated coordinated cations in oxyhalide superionic conductors boost high-rate all-solid-state batteries. J Am Chem Soc 2025; 147: 23170–9. 10.1021/jacs.5c07052 40524353

[50] Zhao F, Zhang S, Wang S et al. Anion sublattice design enables superionic conductivity in crystalline oxyhalides. Science 2025; 390: 199–204. 10.1126/science.adt9678 41066552

[51] Ridley P, Duong G, Ko SL et al. Tailoring chloride solid electrolytes for reversible redox. J Am Chem Soc 2025; 147: 19508–19. 10.1021/jacs.4c14670 40435394 PMC12164357

[52] Lei M, Li B, Liu H et al. Dynamic monkey bar mechanism of superionic Li-ion transport in LiTaCl_6_. Angew Chem Int Ed 2023; 63: e202315628. 10.1002/anie.202315628 38079229

[53] Jun K, Wei G, Yang X et al. Exploring the soft cradle effect and ionic transport mechanisms in the LiMXCl_4_ superionic conductor family. Matter 2025; 8: 102001. 10.1016/j.matt.2025.102001

[54] Zhang SM, Zhao FP, Chang LY et al. Amorphous oxyhalide matters for achieving lithium superionic conduction. J Am Chem Soc 2024; 146: 2977–85. 10.1021/jacs.3c07343 38284994

[55] Zhang SM, Zhao FP, Chen JT et al. A family of oxychloride amorphous solid electrolytes for long-cycling all-solid-state lithium batteries. Nat Commun 2023; 14: 3780. 10.1038/s41467-023-39197-8 37355635 PMC10290651

[56] Li F, Cheng XB, Lu GX et al. Amorphous chloride solid electrolytes with high Li-ion conductivity for stable cycling of all-solid-state high-nickel cathodes. J Am Chem Soc 2023; 145: 27774–87. 10.1021/jacs.3c10602 38079498

[57] Hu L, Wang J, Wang K et al. A cost-effective, ionically conductive and compressible oxychloride solid-state electrolyte for stable all-solid-state lithium-based batteries. Nat Commun 2023; 14: 3807. 10.1038/s41467-023-39522-1 37369677 PMC10300059

[58] Dai T, Wu SY, Lu YX et al. Inorganic glass electrolytes with polymer-like viscoelasticity. Nat Energy 2023; 8: 1221–8. 10.1038/s41560-023-01356-y

[59] Wang G, Zhang S, Wu H et al. Oxychloride polyanion clustered solid-state electrolytes via hydrate-assisted synthesis for all-solid-state batteries. Adv Mater 2024; 37: 2410402. 10.1002/adma.202410402 39529571

[60] Liu H, Liang S, Duan Y et al. Capacity-expanding O/Cl-bridged catholyte boosts energy density in zero-pressure all-solid-state lithium batteries. Natl Sci Rev 2026; 13: nwaf584. 10.1093/nsr/nwaf584 41660574 PMC12875118

[61] Zhou Z, Lu P, Liang S et al. Embedding Fe-based redox chemistry into low-cost oxyhalide solid electrolytes for high-performance all-solid-state batteries. Adv Mater 2026; 38: e72694. 10.1002/adma.72694 41778475

[62] Yue JY, Zhang SM, Wang XY et al. Universal superionic conduction via solid dissociation of salts in van der Waals materials. Nat Energy 2025; 10: 1237–50. 10.1038/s41560-025-01853-2

[63] Li M, Pan H, Liu T et al. All-in-one ionic-electronic dual-carrier conducting framework thickening all-solid-state electrode. ACS Energy Lett 2022; 7: 766–72. 10.1021/acsenergylett.1c02666

[64] Chung S-Y, Bloking JT, Chiang Y-M. Electronically conductive phospho-olivines as lithium storage electrodes. Nat Mater 2002; 1: 123–8. 10.1038/nmat732 12618828

[65] Lu Z-Y, Chen L-T, Hu X et al. Prediction of ground state configurations and electrochemical properties of Li_3_InCl_6_ doped with F, Br, and Ga. Chin Phys Lett 2024; 41: 058201. 10.1088/0256-307X/41/5/058201

[66] Fu J, Su H, Luo J et al. Chemical bond covalency in superionic halide solid-state electrolytes. Angew Chem Int Ed 2025; 64: 202508835. 10.1002/anie.202508835 PMC12322641 40437811

[67] Leube BT, Robert C, Foix D et al. Activation of anionic redox in d^0^ transition metal chalcogenides by anion doping. Nat Commun 2021; 12: 5485. 10.1038/s41467-021-25760-8 34531403 PMC8445930

[68] Cui LF, Zhang S, Ju JW et al. A cathode homogenization strategy for enabling long-cycle-life all-solid-state lithium batteries. Nat Energy 2024; 9: 1084–94.

[69] Hao X, Dong ZL, Ma J et al. A universal solid reaction enabling nanosized Li_2_S in an amorphous matrix for all-solid-state Li-S batteries. J Am Chem Soc 2025; 147: 42184–93. 10.1021/jacs.4c18340 40884457

[70] Minnmann P, Strauss F, Bielefeld A et al. Designing cathodes and cathode active materials for solid-state batteries. Adv Energy Mater 2022; 12: e2201425. 10.1002/aenm.202201425

[71] Huang TP, Zheng Y, Sun DY et al. Multi-level structural modulation enables fast lithium-ion transport in inorganic solid-state batteries. Chem Soc Rev 2026; 55: 433–68. 10.1039/D5CS00895F 41311366

[72] Li QY, Liu H, Ye YS et al. The critical importance of stack pressure in batteries. Nat Energy 2025; 10: 1064–73. 10.1038/s41560-025-01820-x

[73] Yu T, Liu YK, Li HY et al. Ductile inorganic solid electrolytes for all-solid-state lithium batteries. Chem Rev 2025; 125: 3595–662. 10.1021/acs.chemrev.4c00894 39932822

[74] Xiong X, Lin T, Tian C et al. Creep-type all-solid-state cathode achieving long life. Nat Commun 2024; 15: 3706. 10.1038/s41467-024-48174-8 38698026 PMC11065878

[75] Kwak H, Kim JS, Han D et al. Boosting the interfacial superionic conduction of halide solid electrolytes for all-solid-state batteries. Nat Commun 2023; 14: 2459. 10.1038/s41467-023-38037-z 37117172 PMC10147626

[76] Peng D, Li R, Xu K et al. A low-strain lithium cathode material Li_2–2x_Fe_1+x_Cl_4_ for halide-based all-solid-state batteries. ACS Energy Lett 2025; 10: 1421–9. 10.1021/acsenergylett.4c03147

[77] Wang J, Lei X, Guo S et al. Doping strategy in nickel-rich layered oxide cathode for lithium-ion battery. Renewables 2023; 1: 316–40. 10.31635/renewables.023.202200022

[78] Qiu B, Zhou YH, Liang HY et al. Negative thermal expansion and oxygen-redox electrochemistry. Nature 2025; 640: 8060. 10.1038/s41586-025-08765-x 40240602

[79] Ramachandran H, Mu EW, Lomeli EG et al. A formal Fe^III^^/V^ redox couple in an intercalation electrode. Nat Mater 2025; 25: 91–9. 10.1038/s41563-025-02356-x 41094071

[80] Wu R, Bo X, Zhao S et al. Transition metal fluorides as advanced cathodes for lithium/sodium-ion batteries: rational enhancement strategies and underlying electrochemical mechanisms. Adv Funct Mater 2025; 35: 2424603. 10.1002/adfm.202424603

[81] Li L, Jacobs R, Gao P et al. Origins of large voltage hysteresis in high-energy-density metal fluoride lithium-ion battery conversion electrodes. J Am Chem Soc 2016; 138: 2838–48. 10.1021/jacs.6b00061 26847657

[82] Li HS, Huang HQ, Lin YC et al. Unlocking intrinsic voltage plateaus of conversion-type fluoride cathodes via sulfur-mediated dynamic charge redistribution. J Am Chem Soc 2025; 147: 42994–3003. 10.1021/jacs.5c16085 41196568

[83] Wang F, Kim S-W, Seo D-H et al. Ternary metal fluorides as high-energy cathodes with low cycling hysteresis. Nat Commun 2015; 6: 6668. 10.1038/ncomms7668 25808876 PMC4389236

[84] Cui Y, Sukkurji PA, Wang K et al. High entropy fluorides as conversion cathodes with tailorable electrochemical performance. J Energy Chem 2022; 72: 342–51. 10.1016/j.jechem.2022.05.032

[85] Wang Z-W, Xiang S, Luo J-D et al. Achieving 766.5 wh kg^−^^1^ electrode-level energy density via solid-state cathode integrating ultrahigh nickel oxide and lithium iron chloride. Nano Lett 2025; 25: 12930–7. 10.1021/acs.nanolett.5c03012 40814864

[86] Zhang S, Zhao F, Wang S et al. Advanced high-voltage all-solid-state Li-ion batteries enabled by a dual-halogen solid electrolyte. Adv Energy Mater 2021; 11: e2100836. 10.1002/aenm.202100836

[87] Tanibata N, Aizu S, Sasadaira T et al. Redox-level design for high-energy-density chloride electrodes. Adv Energy Mater 2025; 15: e04110. 10.1002/aenm.202504110

[88] Stamenkovic B, Quarez E, Profatilova I et al. Tailoring the fueling capability of halide solid catholyte through composition. Correlation with the underlying redox mechanism. ACS Mater Lett 2024; 6: 4873–80. 10.1021/acsmaterialslett.4c01497

[89] Hu X, Zhang Z, Zhang X et al. External-pressure-electrochemistry coupling in solid-state lithium metal batteries. Nat Rev Mater 2024; 9: 305–20. 10.1038/s41578-024-00669-y

[90] Dong S, Sheng L, Wang L et al. Challenges and prospects of all-solid-state electrodes for solid-state lithium batteries. Adv Funct Mater 2023; 33: 2304371. 10.1002/adfm.202304371

[91] Tan DHS, Meng YS, Jang J. Scaling up high-energy-density sulfidic solid-state batteries: a lab-to-pilot perspective. Joule 2022; 6: 1755–69. 10.1016/j.joule.2022.07.002

[92] Tang W, Wang F, Liang S et al. Polyanion-stabilized amorphous halide electrolytes with low lithium content for all-solid-state lithium batteries. Nat Commun 2026; 17: 3326. 10.1038/s41467-026-69737-x 41764161 PMC13066384

[93] Nie XH, Lei M, Hu JL et al. Boosting Li-ion conductivity of fluoride solid electrolyte by low-temperature molten salt ablation and particle boundary doping. ACS Nano 2024; 18: 30099–112. 10.1021/acsnano.4c12399 39401128

[94] Li X, Liang J, Chen N et al. Water-mediated synthesis of a superionic halide solid electrolyte. Angew Chem Int Ed 2019; 58: 16427–32. 10.1002/anie.201909805 31476261

