# Supplementary material for: A paradigm shift in all-solid-state lithium batteries: halide cathode materials streamlined for multi-electron reactions
Source: Natl Sci Rev. 2026 Jul 16;13(17):nwag438. doi: 10.1093/nsr/nwag438 (PMC13579703; doi:10.1093/nsr/nwag438)
Supplement: Supplementary file 1 [file nwag438_supplemental_file.pdf]

Supporting Information

A paradigm shift in all-solid-state lithium batteries: halide cathode materials streamlined for multi-electron reactions

Yang Luo[1,†], Yuhao Duan[2,3,†], Xiaofei Yang[2,4,*], Xueliang Sun[5,*] and Xianfeng Li[2,4,6,*]

[1]School of Energy and Environmental Engineering, Hebei University of Technology, Tianjin, 300401, China;

[2]Dalian Institute of Chemical Physics, Chinese Academy of Sciences, Dalian 116023, China;

[3]University of Chinese Academy of Sciences, Beijing 100049, China;

[4]Key Laboratory of Long-Duration and Large-Scale Energy Storage, Dalian Institute of Chemical Physics, Chinese Academy of Sciences, Dalian, 116023, China;

[5]Eastern Institute for Advanced Study, Eastern Institute of Technology, Ningbo, 315200, China;

[6]Dalian National Laboratory for Clean Energy, iChEM (Collaborative Innovation Center of Chemistry for Energy Materials), Dalian Institute of Chemical Physics, Chinese Academy of Sciences, Dalian 116023, China

[*]**Corresponding authors.** E-mails: yangxf@dicp.ac.cn; xsun@eitech.edu.cn; lixianfeng@dicp.ac.cn.

[†]Equally contributed to this work.

Table S1. The prices of raw materials on the bulk scale in **Fig. 1d** are estimated from the laboratory-level prices. The estimation method was proposed by Hart et al[1]. The exchange rate of CNY to USD is calculated as 1 USD=7 CNY.

	Purchase quantity Q (g)	Price (USD)	Unit price P (USD kg^{-1})	Unit price in bulk (USD kg^{-1})
LiF, 99.95%, Aladdin	25	3.6	439.4	76.8
	100	14.3	308.4	
	500	71.4	232.5	
	2500	357.1	213.3	
VCl_3, 97%, Adamas Beta	1	22.3	22 300.0	210.4
	5	52.7	10 532.3	
	25	188.4	7535.7	
$TiCl_3$, 78.5% $TiCl_3$-ACl_3, Alfa Aesar	78.5	322.7	4111.0	1513.7
$FeCl_2$, 99.5%, Macklin	1	13.7	13 714.3	569.3
	5	44.0	8800.0	
	25	158.3	6331.4	
	100	484.0	4840.0	
	25	7.4	296.6	
	50	12.1	242.6	
	100	19.4	194.1	
	250	45.7	182.8	
	500	73.1	146.2	
	1000	122.4	122.4	
	2500	266.5	106.6	

Table S2. The prices of commercial raw materials and CAMs in **Fig. 1d**. The exchange rate of CNY to USD is calculated as 1 USD=7 CNY.

Chemical name and description	Data sources	Price (USD kg^{-1})
LiCl, 99%	HuBei Seven Eight Nine Chemical Co., Ltd	14.0
Fe_2O_3, 99%	Wuhan Hengjiu Chemical Co., Ltd	1.6
$FeCl_3$, 98%	Henan Zhengyuan Water Purification Materials Co., Ltd	0.4
$LiFePO_4$		5.00
$LiCoO_2$	https://qqtzj.mysteel.com/	21.4
NCM811, polycrystalline		18.1

Table S3. The price of halide CAMs in **Fig. 1d**. The price of CAMs is estimated based on the proportion of raw material mass.

CAM	Proportion of raw material mass	Price (USD kg^{-1})
Li_3VCl_6	$LiCl : VCl_3 = 0.45 : 0.55$	122.0
Li_3TiCl_6	$LiCl : TiCl_3 = 0.45 : 0.55$	838.9
$LiFeCl_3$	$LiCl : FeCl_2 = 0.5 : 0.5$	305.1
Li_2FeCl_4	$LiCl : FeCl_2 = 0.4 : 0.6$	363.4
$Li_{1.3}Fe_{1.2}Cl_4$	$LiCl : FeCl_2 : FeCl_3 = 0.52 : 0.36 : 0.12$	222.0
LFFOC	$LiF : Fe_2O_3 : FeCl_3 = 0.11 : 0.44 : 0.45$	9.3

Table S4. The energy densities of oxide and halide CAMs in **Fig. 1d**.

CAM	Temperature (°C)	Current density (mA g^{-1})	Energy density (Wh kg^{-1})	Ref.
$LiFePO_4$	25	15.0	450	[2]
NCM811	25	40.0	569	[3]
$LiCoO_2$	25	68.0	539	[4]
Li_3VCl_6	25	9.4	237	[5]
Li_3TiCl_6	25	19.0	452	[6]
$LiFeCl_3$	25	39.0	912	[7]
Li_2FeCl_4	25	12.6	433	[8]
$Li_{1.3}Fe_{1.2}Cl_4$	25	7.5	529	[9]
LFFOC	25	47.6	750	[10]
	60	52.3	1156	

Calculation methods for energy density: The capacity-voltage curves in the references are extracted and integrated to obtain the corresponding energy density at the CAM-level (E_{CAM}). To guarantee that the energy density values are not affected by the anode materials, the voltages corresponding to different anodes are uniformly converted to those corresponding to Li metal anode.

Table S5. The relative density of sulfide and halide CAM/SSE in **Fig. 4a**.

CAM/SSE	Pressure (MPa)	Relative density	Ref.
Li_6PS_5Cl	375	83%	[11]
$Li_{10}GeP_2S_{12}$	350	84.3%	[12]
Li_3TiCl_6	350	86.1%	[6]
Li_3InCl_6	300	88.7%	[3]
$Li_{1.75}ZrCl_{4.75}O_{0.5}$	300	94.2%	[3]
$1.2LiOH\text{-}FeCl_3$	125	98.9%	[13]

REFERENCES

1. Hart PW, Sommerfeld JT. Cost estimation of specialty chemicals from laboratory-scale prices. *Cost Engineering* 1997; **39**: 31-5.
2. Cronk A, Chen YT, Deysher G *et al.* Overcoming the Interfacial Challenges of $LiFePO_4$ in Inorganic All-Solid-State Batteries. *ACS Energy Lett* 2023; **8**: 827-35.
3. Hu L, Wang J, Wang K *et al.* A cost-effective, ionically conductive and compressible oxychloride solid-state electrolyte for stable all-solid-state lithium-based batteries. *Nat Commun* 2023; **14**: 3807.
4. Zhou L, Zuo T-T, Kwok CY *et al.* High areal capacity, long cycle life 4 V ceramic all-solid-state Li-ion batteries enabled by chloride solid electrolytes. *Nat Energy* 2022; **7**: 83-93.
5. Song Z, Dai Y, Wang T *et al.* An active halide catholyte boosts the extra capacity for all-solid-state batteries. *Adv Mater* 2024; **36**: 2405277.
6. Wang K, Gu Z, Xi Z *et al.* Li_3TiCl_6 as ionic conductive and compressible positive electrode active material for all-solid-state lithium-based batteries. *Nat Commun* 2023; **14**: 1396.
7. Zhou X, Jiang M, Duan Y *et al.* Multi-electron transfer halide cathode materials based on intercalation-conversion reaction towards all-solid-state lithium batteries. *Angew Chem Int Ed* 2024; **64**: e202416635.
8. Liu ZT, Zhang GX, Pepas J *et al.* Li_2FeCl_4 as a cost-effective and durable cathode for solid-state li-ion batteries. *ACS Energy Lett* 2024; **9**: 5464-70.
9. Fu JM, Wang CH, Wang S *et al.* A cost-effective all-in-one halide material for all-solid-state batteries. *Nature* 2025; **643**: 111-8.
10. Duan Y, Hussain F, Liu H *et al.* Breaking the conductivity-capacity trade-off in MCl_6 anionic framework: amorphous oxyhalide cathode materials enable ≈1100 Wh Kg^{-1} at cathode-level in all-solid-state lithium batteries. *Adv Mater* 2025; **37**: e13544.
11. Lewis JA, Lee C, Liu Y *et al.* Role of areal capacity in determining short circuiting of sulfide-based solid-state batteries. *ACS Appl Mater Interfaces* 2022; **14**: 4051-60.
12. Wang Y, Hoang B, Hoerauf J *et al.* Hot and cold pressed LGPS solid electrolytes. *J Electrochem Soc* 2021; **168**: 010533.
13. Liu H, Liang S, Duan Y *et al.* Capacity-expanding O/Cl-bridged catholyte boosts energy density in zero-pressure all-solid-state lithium batteries. *Natl Sci Rev* 2026; **13**: nwaf584.
